# Dual Molecular Design toward a Lysosome-Tagged AIEgen and Heavy-Atom-Free Photosensitizers for Hypoxic Cancer Photodynamic Therapy

**DOI:** 10.3390/bios12060420

**Published:** 2022-06-15

**Authors:** Thanh Chung Pham, Thi Thuy Hang Hoang, Yeonghwan Choi, Seongman Lee, Sang-Woo Joo, Gun Kim, Dongwon Kim, Ok-Sang Jung, Songyi Lee

**Affiliations:** 1 Institute for Tropical Technology, Vietnam Academy of Science and Technology, 18 Hoang Quoc Viet, Cau Giay, Hanoi 11300, Vietnam; ptchung@ewha.ac.kr; 2 Division of Chemical Engineering and Materials Science, Ewha Womans University, Seoul 03760, Korea; 3 Department of Information Communication, Materials, and Chemistry Convergence Technology, Soongsil University, Seoul 06978, Korea; hangkt97@soongsil.ac.kr (T.T.H.H.); sjoo@ssu.ac.kr (S.-W.J.); 4 Industry 4.0 Convergence Bionics Engineering, Pukyong National University, Busan 48513, Korea; 96yhchoi@gmail.com; 5 Department of Chemistry, Pukyong National University, Busan 48513, Korea; 1197smlee@gmail.com; 6 Laboratory of Veterinary Pharmacology, College of Veterinary Science and Research Institute for Veterinary Science, Seoul National University, Seoul 08826, Korea; smilesssss@snu.ac.kr; 7 Department of Chemistry, Pusan National University, Busan 46241, Korea; dwkim7459@pusan.ac.kr (D.K.); oksjung@pusan.ac.kr (O.-S.J.)

**Keywords:** photosensitizer, photodynamic therapy, AIE fluorescent probe, lysosome-targeting

## Abstract

To date, a large number of photosensitizers (PS) have introduced heavy atoms to improve the ISC process and ^1^O_2_ generation. However, they often show low efficiency in hypoxic conditions, aggregate states, and turn-off PDT in the dark. Besides that, the toxicity of heavy metals is also concerned. Therefore, we developed lysosome-targeted heavy-metal-free PS (**3S** and **4S**) based on thionated naphthalimide for hypoxic cancer photodynamic therapy (PDT), not only under white light but also in the dark via thermal-induced ^1^O_2_ generation. AIEgen (**3O** and **4O**) were prepared for studying the PDT action of PSs (**3S** and **4S**) in lysosome and aggregate state. We also examined the photophysical properties of AIEgen (**3O** and **4O**) and PS (**3S** and **4S**) by UV–vis absorption, fluorescent emission spectra, and theoretical calculations.

## 1. Introduction

Sulfur-substituted nucleobases have been known for over half a century to be efficient photoactivable medicines with near-unity triplet quantum yields and are still being investigated for oncological uses [1]. Under UVA photoactivation, sulfur-substituted nucleobases have been widely explored for skin cancer treatment, but they generate harmful ROS via both type I and type II photochemical processes [2].

In addition, the effects of aggregation-induced emission (AIE) is a process opposite to the effects of aggregation-caused quenching (ACQ). AIE luminogens (AIEgens) are typically nonemissive in a good solvent but are induced to emit by aggregation [3,4,5,6,7]. Due to the high brightness and photostability of their solutions, AIEgens have emerged as promising fluorescent probes for various biological applications [8,9,10,11]. In particular, among previously reported AIEgens, several AIEgens show efficient photosensitizing ability in the aggregated state, which are beneficial to develop imaging-guided photodynamic therapy (PDT) for cancer treatment [11].

PDT is a medical treatment that has been approved in several countries for the treatment of certain malignancies and other disorders [12,13,14,15,16,17]. PDT requires three ingredients: a light-activated substance (photosensitizer, PS), light, and molecular oxygen [18,19]. The PS is activated to the excited singlet (S_1_) state upon photoexcitation at a specific wavelength; then, the S_1_ state decays back to the ground state, emitting fluorescent or undergoing rapid intersystem crossing (ISC) into the active triplet state (T_1_), producing reactive oxygen species (ROS) via type I and/or type II pathways for cancer photoresponsive treatment [20].

Although PDT has great advantages, it has several limitations for clinical application. One of the problems is dark toxicity from heavy-metal atoms, which are mainly used to accelerate the ISC process [21]. Despite the use of heavy-metal atoms, PDT efficiency is low under hypoxic conditions and turn off in the dark. Unfortunately, cancer cells have many regions that lack oxygen because blood vessels grow slowly but rapidly divide [22]. Moreover, PDT is oxygen-dependent and rapidly consumes intracellular oxygen as PDT progresses. This reduces PDT efficiency in cancer cells. So, it is most important to overcome hypoxia. Another problem is inefficient ROS generation of PS in aggregate condition, which also is one of the typical characteristics of cancer cells and tumors [23]. Many AIE PS have been developed; however, they lack clear evidence of ROS production in aggregate state [24,25]. Herein, we propose that if singlet oxygen could be generated directly in the desired region (in the cancer cells) by a remotely controlled mechanisms, all of these issues would be splendidly avoided.

In this research, we rationally designed and successfully synthesized **4S** composed of naphthalimide and pyridine-2(1*H*)-thione derivatives as a heavy-atom-free PS that can be activated even in hypoxia and aggregate state. The morpholine group in **4S** plays a role in the ability to target the lysosome—an important organelle—and the push–pull effect. As a result, the efficiency of PDT increases and a red shift appears. Type-I and Type-II PDT are possible by substituting sulfur for oxygen in the naphthalimide carbonyl group. The pyridone moiety that reacts with singlet oxygen can form endoperoxide. In the absence of light, endoperoxide from pyridone releases stored singlet oxygen through thermal cycloreversion without other side reactions and returns to its original form of pyridone. During this dark process, it reduces oxygen starvation caused by PDT and allows time for oxygen replenishment. Therefore, the PDT process occurs effectively and continuously in the light/dark cycle. Finally, we demonstrate that the amount of singlet oxygen produced in this way is sufficient for triggering apoptosis in cell cultures.

## 2. Materials and Methods

### 2.1. Synthesis of **4S**

**4O** (1.0 mmol) and Lawesson’s reagent (3.0 mmol) in Toluene (15 mL) was refluxed for 12 h. After solvent was evaporated, it was diluted in DW and extracted 3 times with MC. The organic layer was collected by column chromatography on silica gel using Hexane/Ethyl acetate (4/1) as eluent. The product was dried to afford a red solid of **4S** (yield ~70%). ^1^H NMR (400 MHz, Chloroform-*d*) δ 8.91 (dd, J = 7.7, 1.2 Hz, 1H), 8.86 (d, J = 8.6 Hz, 1H), 8.39 (dd, J = 8.6, 1.3 Hz, 1H), 7.77 (dd, J = 8.7, 1.6 Hz, 1H), 7.67–7.58 (m, 2H), 7.53–7.45 (m, 2H), 7.16 (dp, J = 8.7, 2.1 Hz, 4H), 6.64 (td, J = 6.8, 1.6 Hz, 1H), 5.90 (s, 2H), 4.01 (dd, J = 5.7, 3.4 Hz, 4H), 3.35–3.26 (m, 4H); ^13^C NMR (101 MHz, Chloroform-*d*) δ 192.30, 191.24, 181.26, 156.24, 146.26, 139.97, 139.44, 137.85, 136.53, 135.05, 133.81, 130.52, 130.40, 129.95, 129.24, 126.46, 125.88, 125.44, 125.07, 116.00, 113.86, 66.93, 58.37, 53.34; ESI HRMS *m*/*z* = 514.1077 [M+H]^+^, calc. for C_28_H_23_N_3_OS_3_ = 513.1003.

### 2.2. Cell Experiments

HeLa cells (human cervical cancer cells) were cultured in DMEM (Dulbecco’s Modified Eagle Medium) supplemented with a 10% FBS solution and 1% penicillin-streptomycin (*v*/*v*) and kept in 5% CO_2_ at 37 °C.

### 2.3. Confocal Microscopy Cell Imaging

The HeLa cells were resuspended in confocal dishes to a final density of ~5 × 10^4^ cells/2 mL of the DMEM medium. After overnight culture, the cells were washed twice with DPBS before the fresh culture medium containing **3O** and **4O** (10 μM) was added for 30 min. After washing with DPBS, images were recorded using a confocal microscope equipped with 405-nm excitation and 600-nm emission filters by confocal microscopy Zeiss LSM 900.

HeLa cells were incubated with 5-μM **3S** and **4S**, respectively, and costained with 10 μM DCFH-DA for 30 min. Then, cells were irradiated with a green LED (20 mW/cm^2^, 5 min). After washing with DPBS, fluorescence images were acquired by confocal microscopy.

### 2.4. Cell Viability

Cells were seeded in a 96-well plate to a final density of ∼5 × 10^3^ cells/well with culture media. After overnight culture, HeLa cells were incubated with different concentrations (0–50 μM) of **3S** and **4S** for 1 h. After washing with DPBS, cells were irradiated by a green LED (20 mW/cm^2^, 15 min) and incubated for another 24 h. The samples were subsequently combined with a D-Plus™CCK solution and incubated under normoxia conditions (37 °C, 5% CO_2_) for another 4 h period.

Hypoxic condition was applied by incubation in mixed gas (5% CO_2_, 1% O_2_, 94% N_2_) for 24 h and all media were bubbled with mixed gas beforehand. After incubation with **3S** and **4S** for 1h, green LED was irradiated for 15 min. Then, the cells were incubated for 24 h. The samples were subsequently combined with a D-Plus™CCK solution and incubated under hypoxia conditions (37 °C, 1% O_2_) for another 4 h period.

### 2.5. Theoretical Calculation

Computational calculation is detailed in the supporting information. In the system, crossing rate ($k_{ISC}^{nm})$ between excited singlet state (*S_n_*) to the excited triplet state (*T_m_*) were calculated using Fermi’s Golden rule [26]:

(1) $$k_{ISC}^{nm}=\frac{2\pi }{\hbar }\rho_{FC}{\left|\langle S_{n}\left|H_{SOC}\right|T_{m}\rangle \right|}^{2}$$

where 〈*S_n_*|*H_SOC_*|*T_m_*〉 is the spin–orbit coupling (SOC)-matrix element between *S_n_* and *T_m_* and *ρ_FC_* is Franck–Condon weighted density of states, which was calculated in the framework of the Marcus theory [27]:

(2) $$\rho_{FC}=\frac{1}{\sqrt{4\pi \lambda_{M}k_{B}T}}exp\left[-\frac{{\left(∆E_{ST}+\lambda_{M}\right)}^{2}}{4\pi \lambda_{M}k_{B}T}\right]$$

where ∆*E_ST_*, *T*, *k_B_* and *λ_M_* are singlet-triplet energy gap, temperature, Boltzmann constant, and Marcus reorganization energy, respectively.

## 3. Results and Discussion

### 3.1. Molecular Design, Synthesis Process, and Photophysical Properties

**Scheme 1:**
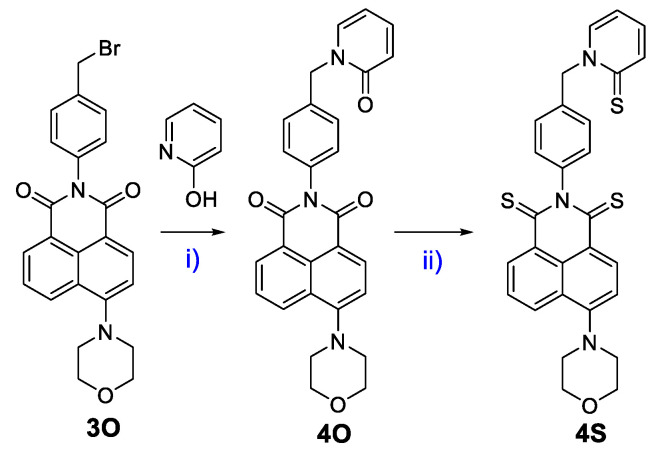
Synthesis of **4S**. (i) K_2_CO_3_, 18-crown-6, KI, Acetone, reflux, N_2_; (ii) and Lawesson’s reagent, Toluene, reflux, 12 h.

**Figure 1:**
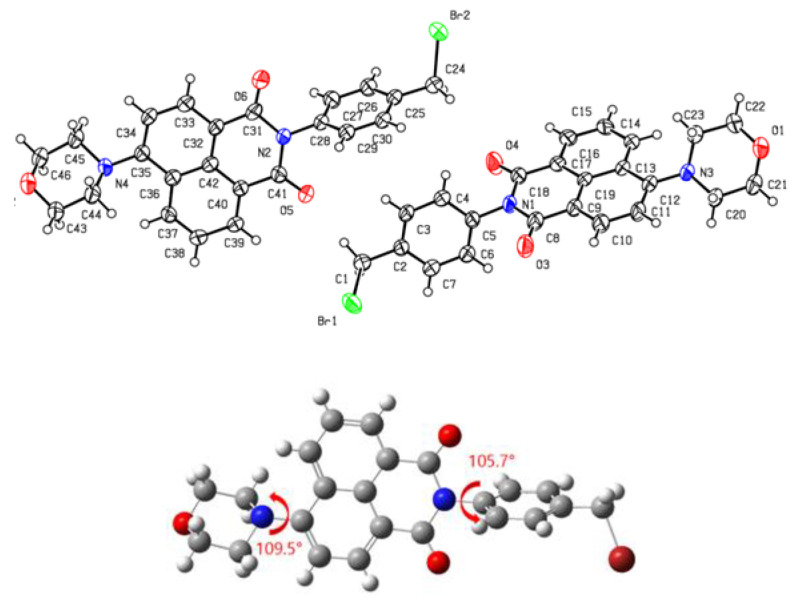
X-ray crystal structure of **3O**.

As in Scheme 1, we prepared **4O** from the reaction of **3O** and 2-hyroxypyridine by adding 18-crown-6 and KI catalyst. Then, the carbonyl group of **4O** was converted to the thiocarbonyl group in the presence of Lawesson’s reagent to form **4S**. We also prepared the thionated form of **3O** (**3S**) by the same method and introduced morpholine to direct the targeting ability of fluorophore and PS toward the lysosome [28]. **4O** and **4S** were conjugated to 2-pyridone and its thiol form, respectively, which are expected to release thermal-induced ^1^O_2_ [29]. All reactions showed high yields (>70%), and products were confirmed by ^1^HNMR, ^13^CNMR, and HR-MS spectra (Supplementary Materials). We observed the ^13^C signal of **3S** and **4S** at about 192 ppm, which confirmed the carbonyl to thiocarbonyl conversion. Notably, the X-ray single-crystal structure of **3O** was recorded (Figure 1 and Figure S17) and found to be similar to the optimized geometry by DFT calculation.

**Figure 2:**
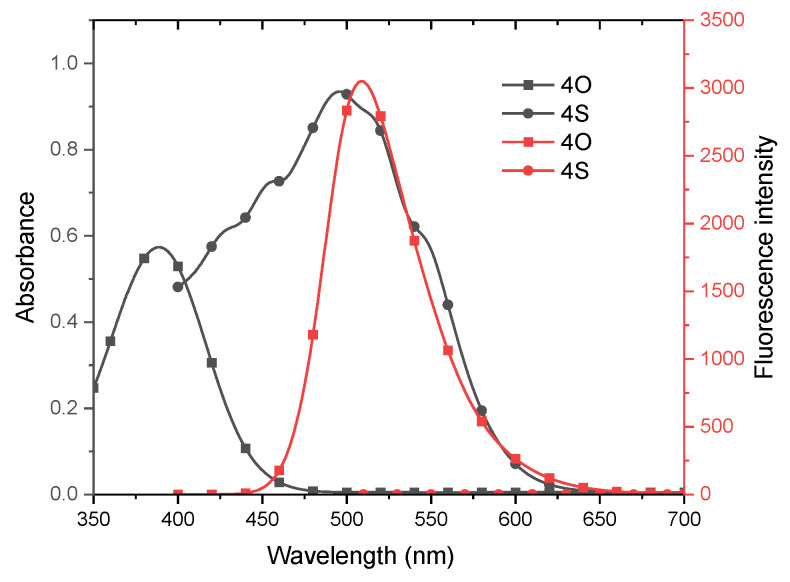
UV–vis absorption (black line) and PL spectra (red line) of **4O** and **4S** in THF (40 µM, λ_ex_ at maximum absorption wavelength, slit 5/5).

Then, we examined the UV–VIS absorption and fluorescent spectra of **3O**, **3S**, **4O**, and **4S** in several solvents (Toluene, THF, and ACN) (Figure 2, Figures S18 and S19). **3O** and **4O**, **3S**, and **4S** show similar UV–Vis absorption bands with peaks near 388 nm and 496 nm, respectively. However, the thionation form shows a higher molar absorption coefficient that corresponds with previous thionated PSs. **3O** and **4O** exhibit a strong green emission (λ_ems_ ~ 509 nm; Φ_F_ > 0.94) with a large stock shift of 120 nm (Table 1), which is very suitable for fluorescent bioimaging. In sharp contrast, **3S** and **4S** do not show fluorescent emission, which predicts a strong ISC process as well as singlet oxygen quantum yield [30]. By computational calculation, the spin–orbit coupling (SOC) of the energy gap between the singlet and triplet states (Δ*E_ST_*) was generated (Tables S4 and S5); then, we calculated the ISC constants (*k_I__SC_*). Due to the thionation, the SOC constants of **4S** and **3S** are significantly higher than that of **4O** and **3O**, respectively. Besides that, the energy gap between the singlet and triplet states of the thiocarbonyl form is also smaller than that of the corresponding the carbonyl form, leading to much larger single ISC constants (*k_ISC_*) of each singlet–triplet pathway. As a result, the total ISC constants of **4S** and **3S** are significantly higher than those of **4O** and **3O**, respectively. The increased ISC process of thionation facilitates the popular triplet states of **4S** and **3S**, which were confirmed by strong ^1^O_2_ generation quantum yield (Φ_Δ_ = 0.50 and 0.56, respectively). Additionally, the total ISC constant of **3S** (9.7 × 10^12^), higher than that of **4S** (5.6 × 10^12^), completely corresponds with the higher ^1^O_2_ generation quantum yield, which confirms the well-matched experimental/theoretical results.

**Figure 3:**
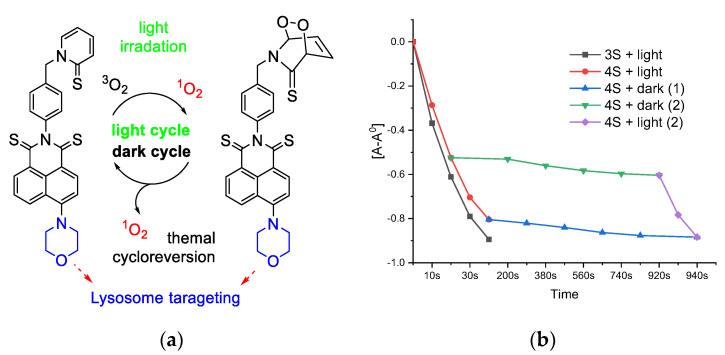
(**a**) Proposed mechanism of light/dark controlled release of lysosome-targeted heavy-metal-free PSs; (**b**) the decreased absorbance of DPBF (^1^O_2_ generation detector) (50 µM) in the presence of **3S** (20 µM) and **4S** (20 µM) under light/dark conditions.

The ROS generation of **4S** was caused not only by ^3^O_2_ → ^1^O_2_ conversion but also by biomolecules → O_2_^•−^ production under green light irradiation. The fluorescent emission of dihydroethidium (O_2_^•−^ probe) in degassed DW (10% fetal bovine serum) increased in the presence of **4S** and white light during 20 min (Figure S20e,f), which confirmed the ROS generation in aggregate state and type I caused by the sensitive excited C=S bond and amino groups [30]. The type I ROS generation, which helps the PSs work well in the hypoxic condition, has been desired in recent years. On the other hand, the pyridine-2(1*H*)-thione introduced in **4S** can store the generated ^1^O_2_ in the light irradiation phase and thermally release it in the dark phase (Figure 3a). In the presence of **4S**, the UV–Vis absorption spectra of the ^1^O_2_ detector (DPBF) quickly decay under white-light irradiation and slowly decrease in the dark and 37 °C (Figure 3b). After the 940 s light/dark condition, its decreasing of the UV–Vis absorbance of DPBF was similar to that of **3S** in 40 s light irradiation. Maintaining ^1^O_2_ release facilitates the killing of cancer in the dark. Besides that, C=S boned-based chemosensors have been developed for detecting OCl^−^ [31]. However, **3S** and **4S** shows stability in the presence of various ROS types and pH ranges, which is observed by negligible change in UV–Vis absorption and FL emission spectra (Figures S22 and S23). The stability of PS facilitates an efficient PDT action in cancer cell.

### 3.2. In Vitro Experiment

#### 3.2.1. Cell Imaging

**Figure 4:**
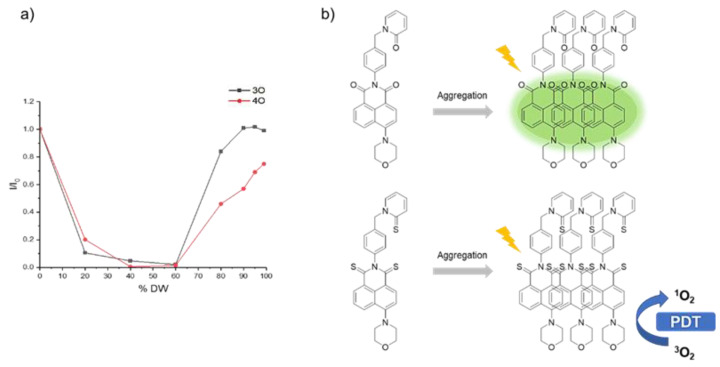
(**a**) Fluorescent emission of **3O** and **4O** at 508 nm in THF/DW (0–99%); (**b**) proposed scheme of AIE/PDT active fluorescent probes.

**Figure 5:**
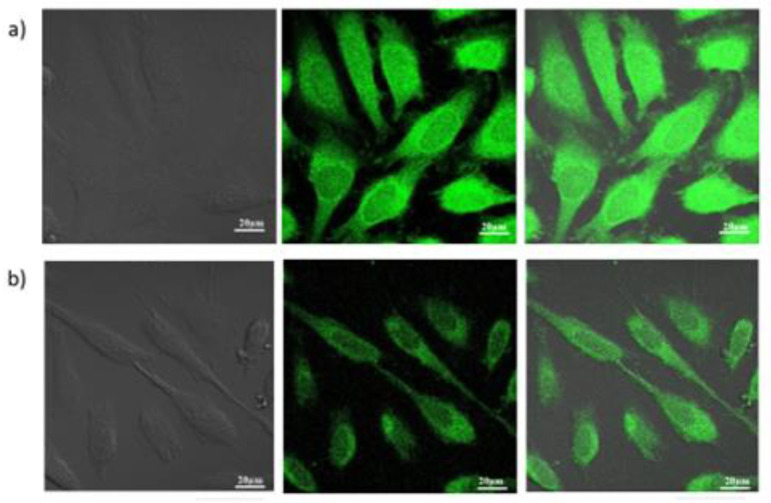
Fluorescent image of HeLa cell after incubation with (**a**) **3O** and (**b**) **4O**.

**Figure 6:**
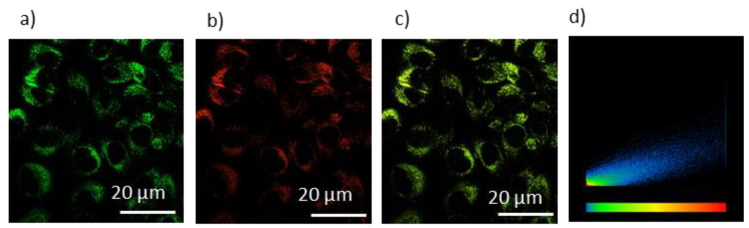
Hela cells costained with (**a**) 10 μM **4O** (λ_ex_ = 405 nm, λ_em_ = 600 nm) for 30 min and (**b**) 50 nM LysoTracker deep red for 1 h (λ_ex_ = 647 nm, λ_em_ = 655 nm) in PBS and fluorescence images acquired by confocal microscopy. (**c**) An overlay. (**d**) Colocalization analysis plot of the LysoTracker and **4O**. Pearson’s overlap coefficient, R_P_ = 0.82.

Due to strong fluorescent emission (Φ_F_ > 0.94), **3O** and **4O** may be usable for cell imaging applications. We further examined their emissions in the aggregate state. Interestingly, the fluorescent intensity of **3O** and **4O** was recovered with an increasing water content (80–99%) in the THF solution (Figure 4 and Figure S21), which indicates AIE properties because of the restricted intramolecular rotation between the naphthalimide and morpholine groups—that is, the naphthalimide and benzene ring (Figure 1b). This also provides alternative evidence of ROS generation of **3S** and **4S** in aggregate state due to their similar structures. Encouraged by the excellent emission, we further evaluated the cellular uptake of **3O** and **4O** in HeLa living cells by confocal fluorescent imaging. **3O** exhibited a brighter green emission than did **4O** because of a stronger AIE effect (Figure 4). The cell imaging and viability of HeLa was retained in the presence of **3O** and **4O** (0–50 µM) (Figure 5 and Figure S22a,c), indicating their excellent biosafety and biocompatibility. On the other hand, their structures introduced morpholine-lysosome targeting groups, which may be useful as a lysosome-targeted fluorescent probe [28]. The emission signal of parent **3O** and **4O** overlapped well with that of commercial LysoTracker deep red with a high Pearson’s coefficient value (R_P_ = 0.85 and 0.82, respectively) (Figure 6 and Figure S25), suggesting specific localization of **3O** and **4O** in lysosomes.

#### 3.2.2. PDT in Normoxia and Hypoxia

**Figure 7:**
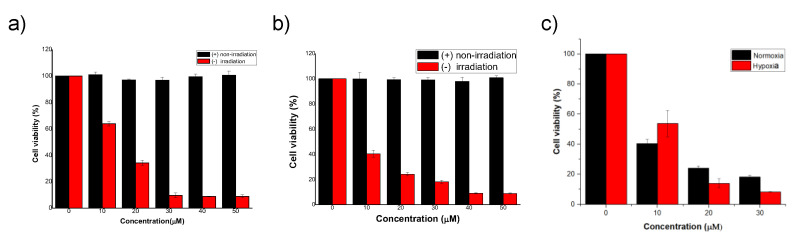
Viability of HeLa cells in the presence of (**a**) **3S** and (**b**) **4S** with and without light irradiation; (**c**) viability of HeLa cells in the presence of **4S** with light irradiation under normoxia and hypoxia.

**Figure 8:**
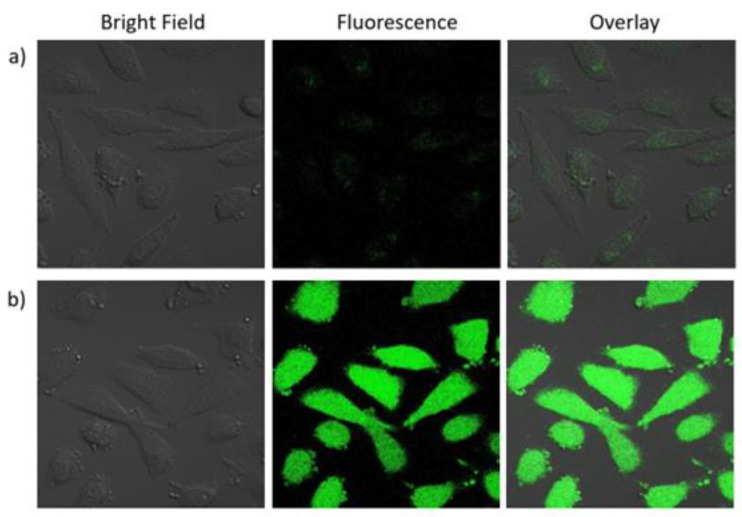
Fluorescent image of HeLa cell after incubation with (**a**) **3O** and (**b**) **4O**. Fluorescent image of HeLa cells in the presence of DCFH-DA and **4S** (**a**) before and (**b**) after irradiation.

To demonstrate their potential for photoresponsive therapy, we explored the anticancer efficacy of **3S** and **4S** toward HeLa cells via methyl thiazolyl tetrazolium (MTT) assays. The viability of HeLa cells was retained when **3S** and **4S** (0–50 µM) were increased without light irradiation (Figure S22b,d), indicating the negligible dark cytotoxicity. Under light irradiation, the viability decreased in the presence of **3S** and **4S** from 0 to 50 µM (Figure 7a,b). At the 10 µM concentration, the viability of HeLa cells toward **3S** and **4S** was 63.9% and 40.5%, respectively. Due to the specific localization of **3O** and **4O** in lysosomes, we speculate that **3S** and **4S** with highly similar structures will destroy cancer cell lysosomes under irradiation to induce severe apoptotic cell death [28]. Interestingly, **4S** showed high photocytotoxicity not only in normoxia but also in hypoxia, with a similar cell viability (Figure 7c). The ROS generation of PSs in HeLa cells during PDT was confirmed by turn-on of fluorescent emission of the DCHF-DA detector (Figure 8). The efficient PDT action of **4S** in both conditions can be explained by the dual Type I + II ROS generation because of the efficient formation of triplet states in aggregate state [32], introduction of sensitive excited C=S bond and amino groups [30], and thermal-induced releasing of ^1^O_2_ in the dark [29].

## 4. Conclusions

We examined the photophysical properties and excited states of fluorophore (**3O** and **4O**) and PSs (**3S** and **4S**) by joint experimental/theoretical studies. **3O** and **4O** showed intense fluorescent visualization in HeLa cells due to AIE effect, whereas **4S** PSs exhibited absorbing/releasing cycloreversion of ^1^O_2_ and strong ROS generation even in the aggregate condition because of the strong ISC process and sensitivity of the excited C=S bond and amino groups. **4S** can exhibit a lysosome-targeted PDT efficiency in both normoxic and hypoxic environments.

## Figures and Tables

**Table 1:** Photophysical and photosensitizing properties of **4O** and **4S**.

	λ_abs_*^a^* (nm)	ԑ × 10^3^ (M^−1^ cm^−1^)	λ_ems_ *^a^* (nm)	∆v *^b^* (nm)	Փ_F_ *^a,c^*	E_g_ *^d^* (eV)	*k_ISC_* *^e^*	Փ_∆_ *^f^*
**3O**	388	11.96	509	121	0.94	3.58	5.1 × 10^10^	-
**3S**	496	20.20	-	-	-	2.47	9.7 × 10^12^	0.56
**4O**	389	13.48	509	120	0.98	3.40	3.9 × 10^8^	-
**4S**	496	23.37	-	-	-	2.29	5.6 × 10^12^	0.50

*^a^* in THF. *^b^* Stock shift. *^c^* Fluorescence quantum yield evaluated with Rhodamine 6G as the standard (Փ_F_ = 0.94 in Ethanol). *^d^* Eg, the energy difference between the HOMO and LUMO levels. *^e^* $k_{ISC}^{\;}=\;\frac{2\pi }{\hbar }\rho_{FC}{\left|\langle S_{n}\left|H_{SOC}\right|T_{m}\rangle \right|}^{2}$. *^f^* Singlet oxygen quantum yield was determined with respect to Ru(bpy)_3_^2+^ (Φ_∆_ = 0.57 in ACN)—not observed).

## Data Availability

Not applicable.

## Supplementary Materials

The following supporting information can be downloaded at: https://www.mdpi.com/article/10.3390/bios12060420/s1, Figures S1–S16: NMR and Mass spectra data; Table S1: Crystal data and structure refinement for **3O**; Figure S17: X-ray result; Figures S18–S21: UV–vis and Fluorescence spectra analysis; Tables S2–S5: Computational calculation results; Figure S22: Cell experiment results.
