# Supplementary material for: Dual Molecular Design toward a Lysosome-Tagged AIEgen and Heavy-Atom-Free Photosensitizers for Hypoxic Cancer Photodynamic Therapy
Source: Biosensors (Basel). 2022 Jun 15;12(6):420. doi: 10.3390/bios12060420 (PMC9221524; doi:10.3390/bios12060420)
Supplement: Supplementary file 1 [file biosensors-12-00420-s001.zip › biosensors-1731391-supplementary.pdf]

Supporting Information for "Dual molecular design toward a lysosome-tagged AIEgen and heavy-atom-free photosensitizers for hypoxic cancer photodynamic therapy"

Thanh Chung Pham,[d,e,+] Thi Thuy Hang Hoang,[c,+] Yeonghwan Choi,[b] Seongman Lee[b] Sang-Woo Joo,[c] Gun Kim,[f] Dongwon Kim,[g] Ok-Sang Jung,[g] Songyi Lee*,[a,b]

[a]Department of Chemistry, Pukyong National University, Busan 48513, Korea;

[b]Industry 4.0 Convergence Bionics Engineering, Pukyong National University, Busan 48513, Korea;

[c]Department of Information Communication, Materials, and Chemistry Convergence Technology, Soongsil University, Seoul 06978, Republic of Korea

[d]Institute for Tropical Technology, Vietnam Academy of Science and Technology, 18 Hoang Quoc Viet, Cau Giay, Hanoi, Vietnam.

[e]Division of Chemical Engineering and Materials Science, Ewha Womans University, Seoul 03760, Korea.

[f]Laboratory of Veterinary Pharmacology, College of Veterinary Science and Research Institute for Veterinary Science, Seoul National University, Seoul 08826, Korea.

[g]Department of Chemistry, Pusan National University, Busan 46241, Korea

[+]These authors contributed equally.

slee@pknu.ac.kr

Contents

1. NMR and Mass spectra data……………………………………………………………………..Page S6
2. X-ray result……………………………………………………………………………………Page S12
3. UV–vis and Fluorescence spectra analysis……………………………………………Page S13
4. Computational calculation result………………………………………………………………Page S16
5. Cell experiments Data……………………………………………………………………… Page S20
6. References……………………………………………………………………………………… Page S21

1. Experimental data

1.1. General consideration

All reagents and organic solvents used in the synthesis were obtained from Aldrich, TCI (South Korea) and used without further purification. Flash chromatography was carried out on silica gel (230-400 mesh), followed by the determination of ^{1}H and ^{13}C NMR spectra using a Bruker Avance 400 MHz spectrometer. Mass spectra were obtained using a maXis-HD (Bruker). UV absorption spectroscopy measurements were carried out on a V-730 UV–Visible Spectrophotometer (Jasco) at room temperature. Fluorescence emission spectra were obtained using an F-7000 Fluorescence Spectrophotometer (Hitachi High-Tech).

1.2. Synthesis process

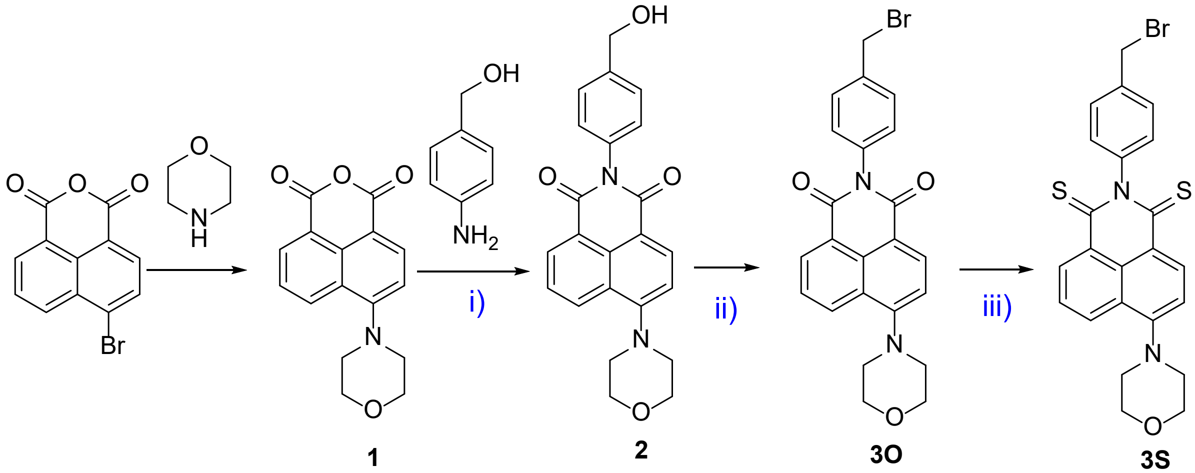

1 2 3O 3S

Scheme S1. The synthesis process of **4O**. i) DBU, DMF, reflux, 12 h; iii) PBr_3, DCM, RT; iii) Lawesson's reagent, Toluene, reflux.

Synthesis of **1** followed the procedure of the previous report.[1]

Synthesis of **2**: **1** (1.0 mmol), 4-Aminobenzyl alcohol (1.0 mmol), and 1,8-Diazabicyclo[5.4.0]undec-7-ene (1.2 mmol) were dissolved in DMF (15 mL). The mixture was stirred at 95 °C for 24 h. After being cooled to room temperature, ice-cold water was added to the reaction mixture, which was filtered and washed by the ice-cold water to obtain crude product. The crude product was purified by column chromatography on silica gel using dichloromethane/ethyl acetate (2/1) as eluent. The product was dried to afford a **2** (yield ~60 %). ^{1}H NMR (400 MHz, Acetone-d_6) δ 8.62 (dd, J = 8.5, 1.2 Hz, 1H), 8.52 (dd, J = 7.2, 1.2 Hz, 1H), 8.46 (d, J = 8.0 Hz, 1H), 7.82 (dd, J = 8.5, 7.3 Hz, 1H), 7.50 - 7.46 (m, 2H), 7.41 (d, J = 8.1 Hz, 1H), 7.31 (d, J = 8.4 Hz, 2H), 4.70 (d, J = 6.0 Hz, 2H), 3.99–3.95 (m, 4H), 3.32–3.27 (m, 4H); ^{13}C NMR (101 MHz, Chloroform-*d*) δ 164.74, 164.25, 156.07, 141.46, 134.98, 133.10, 131.74, 130.60, 130.40, 128.91, 127.95, 126.36, 126.04, 123.50, 117.21, 115.13, 67.06, 65.12, 53.5.

Synthesis of **3O**: **2** (1.0 mmol) and PBr_3 (1.3 mmol) were dissolved in dichloromethane (20 mL). The mixture was stirred at room temperature for 12 h under N_2 atmosphere. The solution was washed with $NaHCO_3$ solution and extracted with dichloromethane. The organic phase was dried with Na_2SO_4 and evaporated under reduced

pressure to obtain crude product as green solid. The crude product was purified by column chromatography on silica gel using dichloromethane/methanol (150/1) as eluent. The product was dried to afford a green solid of **3O** (yield ~80 %). ^{1}HNMR (400 MHz, Acetone-d_6) δ 8.64–8.61 (m, 1H), 8.53–8.50 (m, 1H), 8.49–8.44 (m, 1H), 7.85–7.79 (m, 1H), 7.62–7.58 (m, 2H), 7.43–7.40 (m, 1H), 7.40–7.36 (m, 2H), 4.73 (s, 2H), 4.00–3.94 (m, 4H), 3.32–3.27 (m, 4H); ^{13}CNMR (101 MHz, Chloroform-d) δ 133.12, 130.63, 130.15, 129.19, 126.04, 115.13, 67.05, 53.54, 32.76; ESI HRMS m/z = 473.0475 $[M+Na]^+$, calc. for $C_{23}H_{19}BrN_2O_3$ = 450.0579.

Synthesis of **3S**: **3O** (1.0 mmol) and Lawesson's reagent (3.0 mmol) in Toluene (15 mL) were refluxed for 24 h under N_2 atmosphere. Afterwards, the solvent was evaporated under reduced pressure. The residue was purified by column chromatography on silica gel using dichloromethane/n-hexane (4/1) as eluent. The product was dried to afford a black solid of **3S** (yield ~10 %). ^{1}HNMR (400 MHz, Acetone-d_6) δ 8.90 - 8.87 (m, 1H), 8.82 (d, J = 8.5 Hz, 1H), 8.60 (dd, J = 8.4, 1.2 Hz, 1H), 7.72 (dd, J = 8.3, 7.7 Hz, 1H), 7.59 - 7.55 (m, 2H), 7.36 (d, J = 8.6 Hz, 1H), 7.24–7.19 (m, 2H), 4.71 (s, 2H), 3.99–3.95 (m, 4H), 3.40–3.37 (m, 4H); ^{13}CNMR (101 MHz, Chloroform-d) δ 192.40, 156.14, 145.05, 141.00, 139.46, 137.89, 130.58, 130.28, 129.37, 128.61, 126.45, 125.89, 125.48, 125.16, 115.99, 66.94, 53.34, 29.79, 28.85; ESI HRMS m/z = 505.0005 $[M+Na]^+$, calc. for $C_{23}H_{19}BrN_2OS_2$ = 482.0122.

Synthesis of **4O**: **3O** (1.0 mmol), 2-Hydroxypyridine (1.0 mmol), and Potassium carbonate (3.0 mmol) were dissolved in Acetone (20 mL). After adding a catalytic amount of 18-crown-6 and KI, the reaction mixture was refluxed for 16 h under N_2 atmosphere. After completion of the reaction, the reaction mixture was extracted with dichloromethane. The organic phase was dried with Na_2SO_4 and evaporated under reduced pressure to obtain crude product as green solid. The crude product was purified by column chromatography on silica gel using dichloromethane/methanol (50/1) as eluent. The product was dried to afford a green solid of AK6 (yield ~70 %). ^{1}HNMR (400 MHz, Acetone-d_6) δ 8.61 (dd, J = 8.5, 1.5 Hz, 1H), 8.50 (dd, J = 7.3, 1.3 Hz, 1H), 8.45 (d, J = 7.8 Hz, 1H), 7.84–7.78 (m, 1H), 7.73 (dd, J = 6.8, 2.2 Hz, 1H), 7.49 (d, J = 8.2 Hz, 2H), 7.43–7.37 (m, 2H), 7.36–7.32 (m, 2H), 6.42 (dd, J = 9.1, 1.0 Hz, 1H), 6.24–6.15 (m, 1H), 5.23 (s, 2H), 3.98–3.95 (m, 4H), 3.30–3.27 (m, 4H); ^{13}CNMR (101 MHz, Chloroform-d) δ 164.61, 164.11, 162.77, 139.57, 137.37, 136.83, 133.11, 131.74, 130.62, 130.39, 129.39, 129.34, 126.35, 126.03, 123.46, 121.40, 117.14, 115.12, 106.45, 67.05, 53.54, 51.47; ESI HRMS m/z = 488.1586 $[M+Na]^+$, calc. for $C_{28}H_{23}N_3O_4$ = 465.1689.

1.3. Photophysical measurement

The photoluminescence quantum yield (Φ_F) was determined according to the following equation:

$$\Phi_s = \Phi_{ref} \times \left(\frac{Grad_s}{Grad_{ref}}\right) \times \left(\frac{\eta_s}{\eta_{ref}}\right)^2$$

where the subscripts *ref* and *s* denote reference and test, respectively; Φ is the fluorescence quantum yield; Grad is the gradient from the plot of integrated fluorescence intensity and absorbance; and η is the refractive index of the solvent. Rhodamine 6G (Φ_F = 0.94 in ethanol) was used as a reference.[2]

1.4. ROS generation measurements

Calculation of relative singlet oxygen quantum yields (Φ_Δ) was performed by following the literature.[3] Relative singlet oxygen quantum yields were calculated and compared to Rose Begal (Φ_Δ = 0.54 in ACN).[4] The absorption of DPBF at 414 nm was recorded every 1 or 2 seconds to obtain the photosensitizing process' decay rate. Measurements were performed using a green LED light source (5 $mW.cm^{-2}$). 1,3-Diphenylisobenzofuran (DPBF, as singlet oxygen trap, abs ~ 1.20) and photosensitizers (abs ~ 0.2) were placed in a cuvette containing air-saturated organic solvents, and the solutions were kept in the dark until the absorbance reading was stable, followed by continuous light irradiation. The $^1O_2$ quantum yields of the BDP dyes were calculated according to the following equation:

$$\Phi_{PS} = \Phi_{ref} \times \frac{m_{PS}}{m_{ref}} \times \frac{F_{ref}}{F_{PS}}$$

where *PS* and *ref* for test and reference, respectively. *m* is the slope of the change in absorbance of DPBF at absorbance maxima with the irradiation time. *F* is the absorption correction factor, which is given as $F = 1\text{-}10^{-OD}$.

The $O_2^{\bullet-}$ production of **4S** in DW (10 % Fetal bovine serum) was investigated using dihydroethidium (DHE) as a probe.

1.5. Computational calculation detail

The DFT calculations of the molecules were performed using the Gaussian 09 program package. Geometry optimizations of the molecule were performed using the B3LYP hybrid functional with 6-31+G(2d,p) basis set. After optimizing structures, vibrational frequencies were computed to ensure that there were no imaginary frequencies. Optical excitation energies were calculated with various functionals using time-dependent DFT (TD-DFT) with the 6-31+G(2d,p), def-2-TZVP, cc-pVDZ basis sets in THF solvent. The solvent was modeled by the polarizable continuum model (PCM) using the integral equation formalism variant (IEFPCM), as implemented in Gaussian 09. Our calculated excitation energies using the M06 functional with cc-pVDZ were nearest to the experimental data. The spin–orbit coupling (SOC) constants, $\langle S_n|H_{SOC}|T_m\rangle$, between singlet and triplet states for a given molecule were calculated. The detailed calculation method for the SOC constants was performed by pySOC program.[5] We calculated the ISC rate from the S_1, S_2, S_3, S_4 states to all of the triplet excited states (T_m) with energy less than that of S_n.

2. Hypoxia in vitro experiment

HeLa cells were cultured in 96-well plate in mixed gas (5% CO_2, 1% O_2, 94% N_2) (5000 cells/well). Hela cells were then treated with different concentrations of **3S** and **4S** (from 0-50 µM) for 1 h. After washing with DPBS, cells were irradiated by a green LED (20 mW/cm^2, 15 min) and incubated for another 24 h. The cells were rinsed very carefully to remove excess chemicals, treated with D-PlusTM CCK solution, and incubated in hypoxia condition (37 °C, 5% CO_2) for another 4 h period.

3. Results

3.1. NMR and Mass spectra

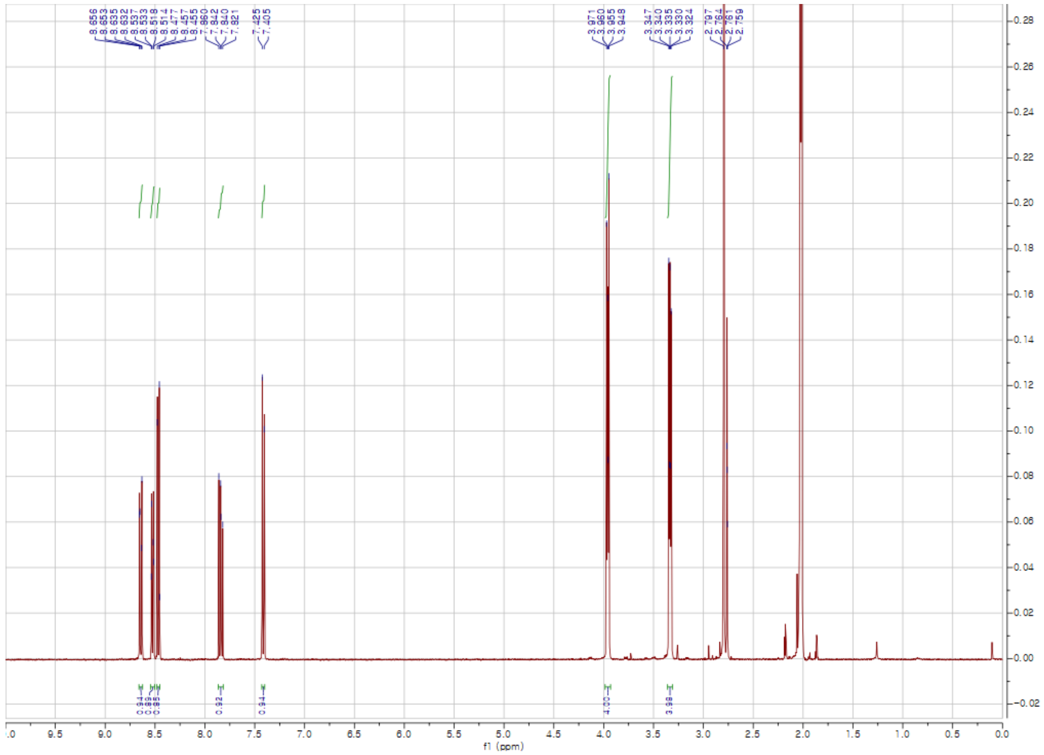

Figure S1. ^{1}H-NMR spectra of **1** in Acetone-d_6.

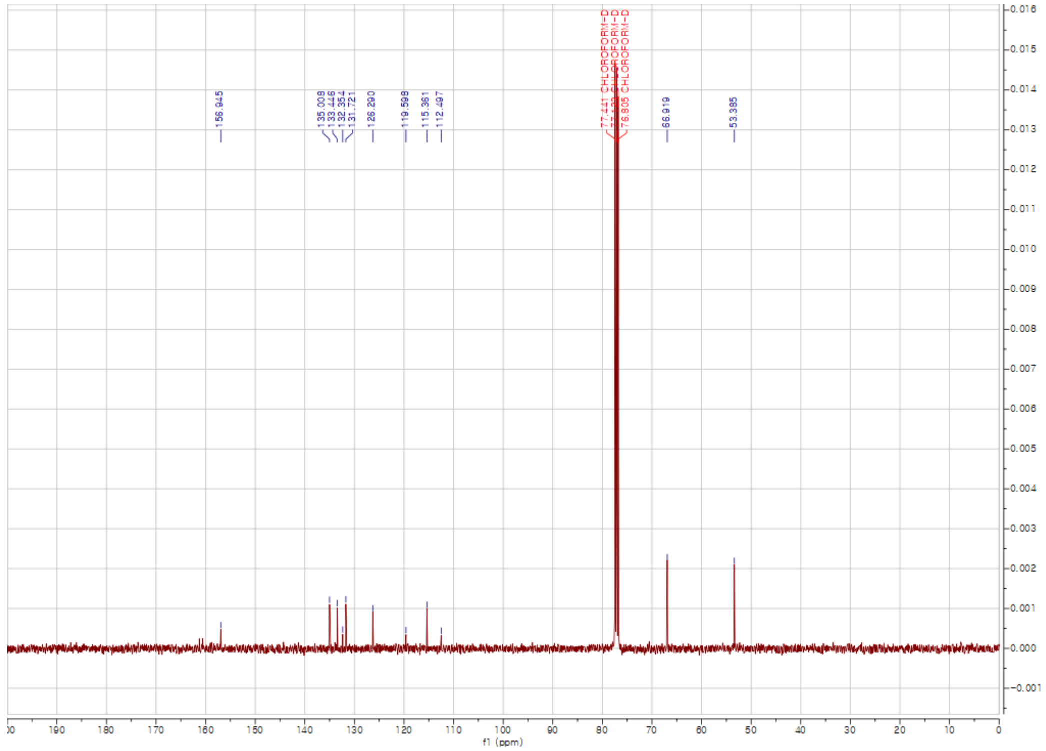

Figure S2. ^{13}C-NMR spectra of **1** in $CDCl_3$.

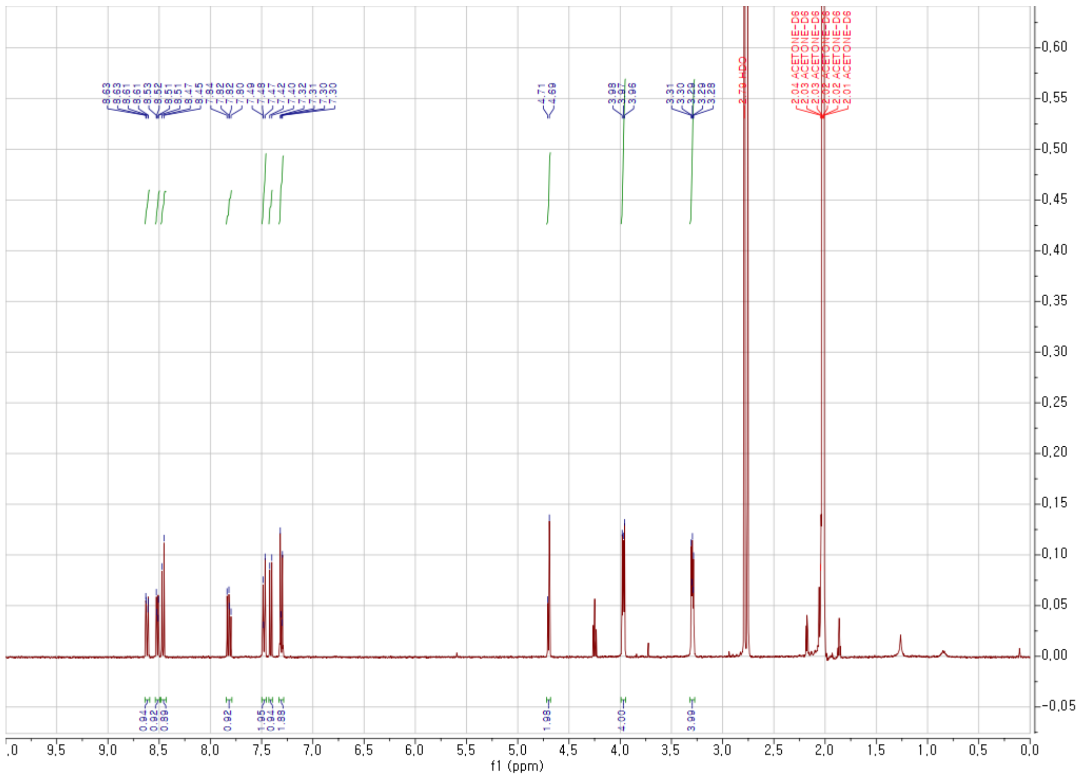

Figure S3. ^{1}H-NMR spectra of **2** in Acetone-d_6.

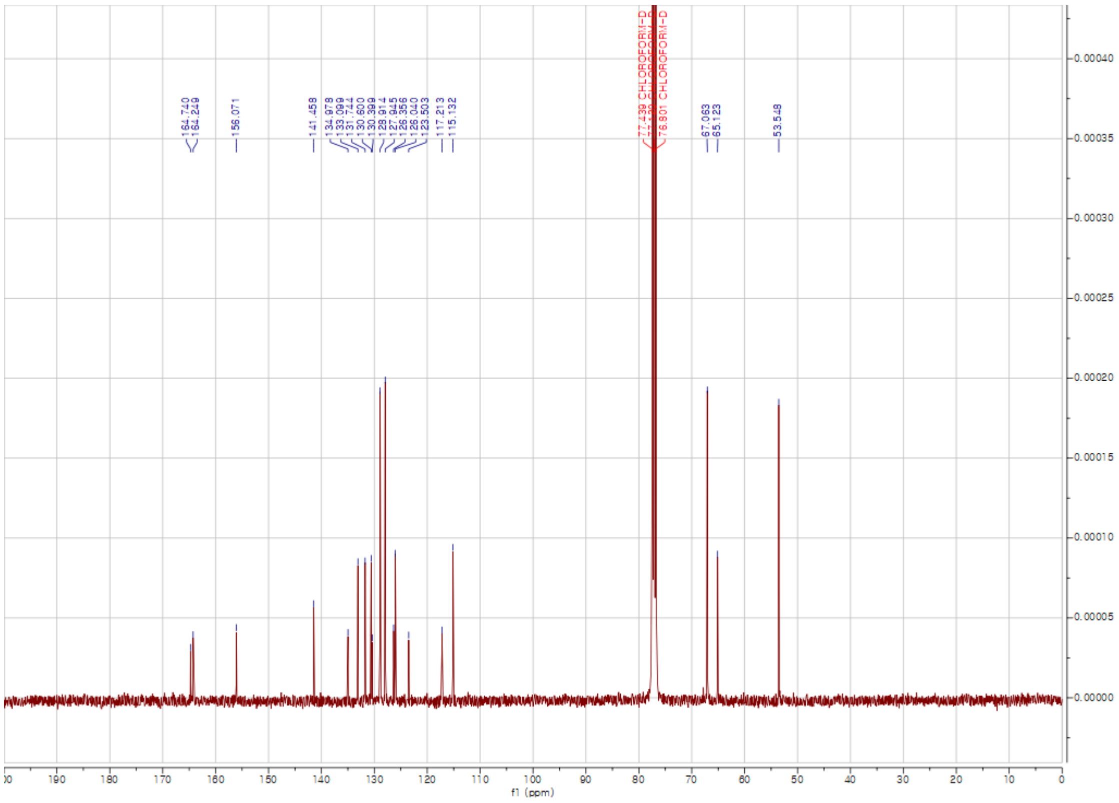

Figure S4. ^{13}C-NMR spectra of **2** in $CDCl_3$.

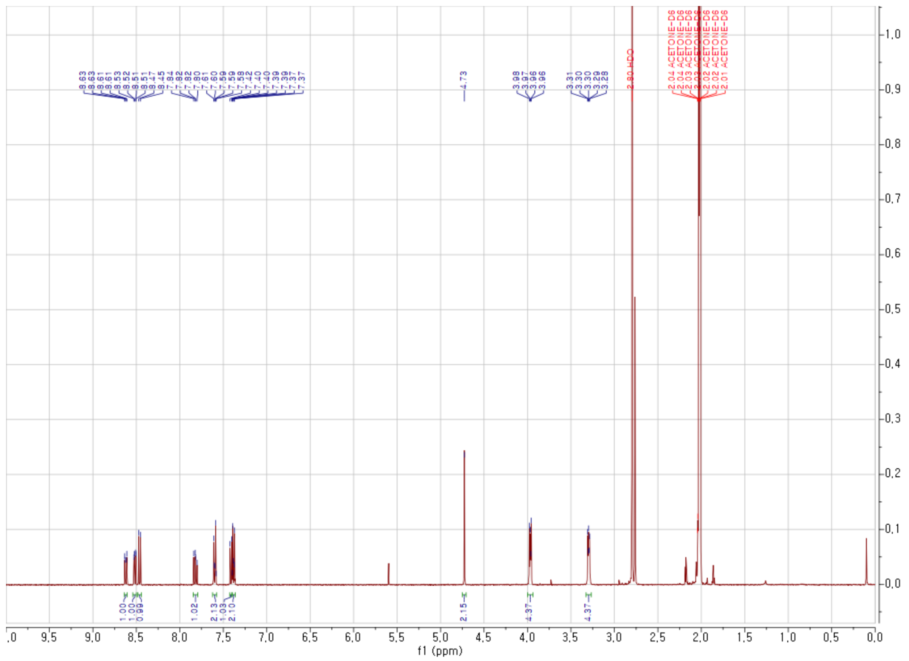

Figure S5. ^{1}H-NMR spectra of **3O** in Acetone-d_6.

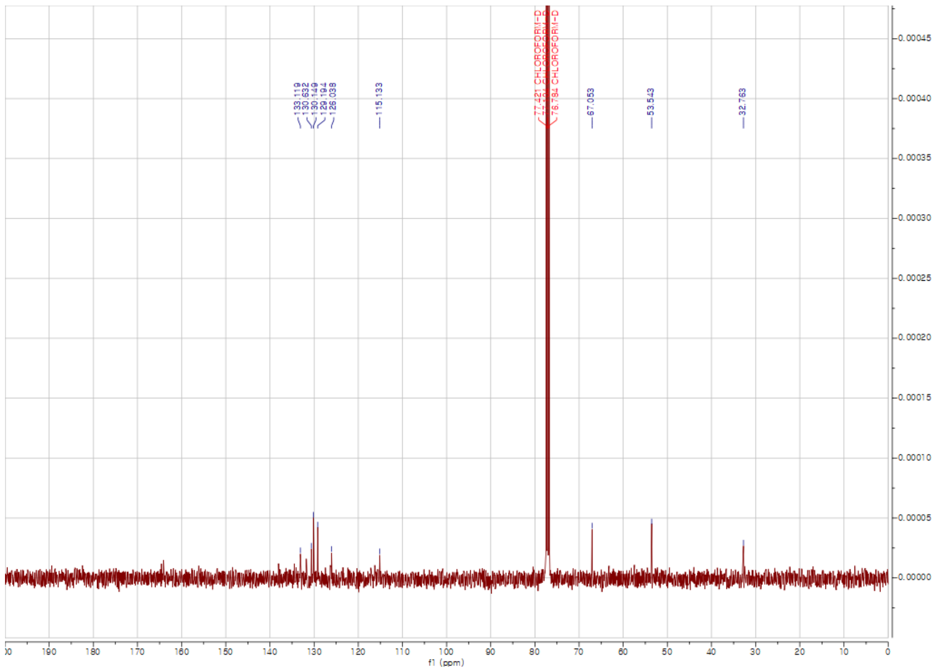

Figure S6. ^{13}C-NMR spectra of **3O** in $CDCl_3$.

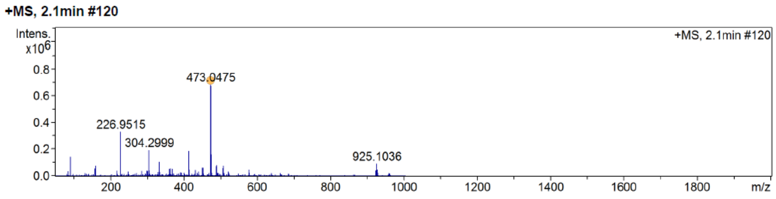

Meas. m/z	#	Ion Formula	m/z	err [ppm]	mSigma	# mSigma	Score	rdb	e⁻ Conf	N-Rule
473.0475	1	C23H19BrN2NaO3	473.0471	-0.9	13.0	1	100.00	14.5	even	ok

Figure S7. Mass spectra of **3O.**

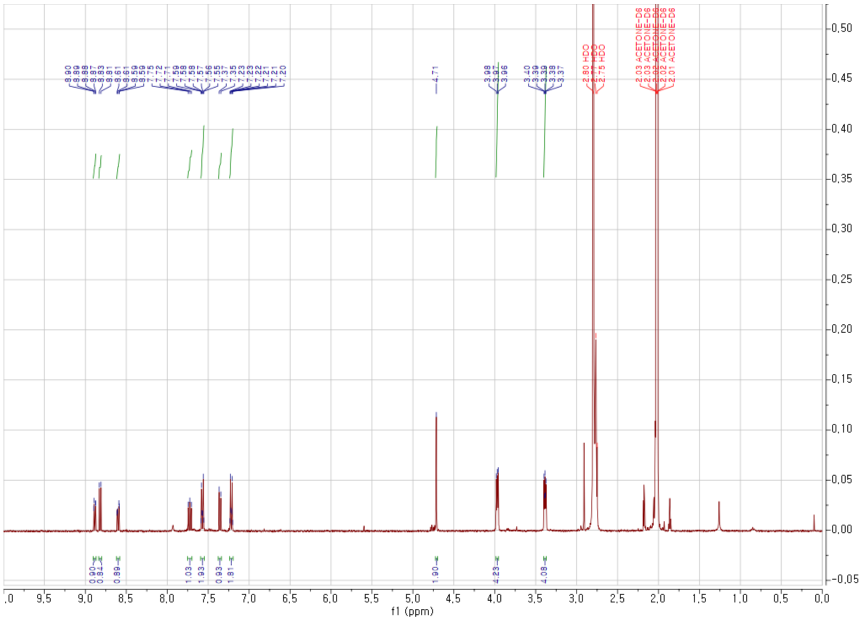

Figure S8. ^{1}H-NMR spectra of **3S** in Acetone-d_6.

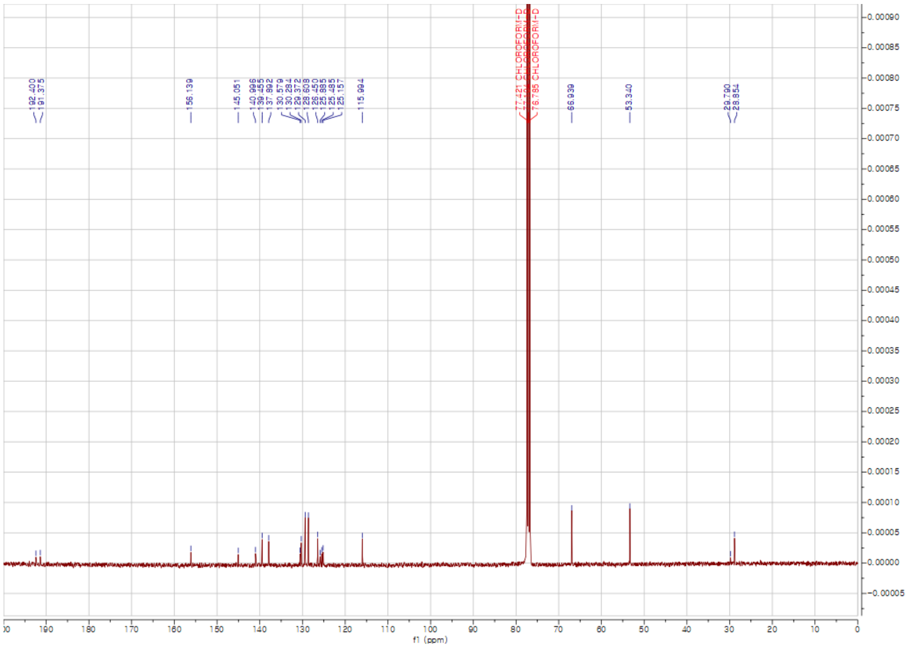

Figure S9. ^{13}C-NMR spectra of **3S** in $CDCl_3$.

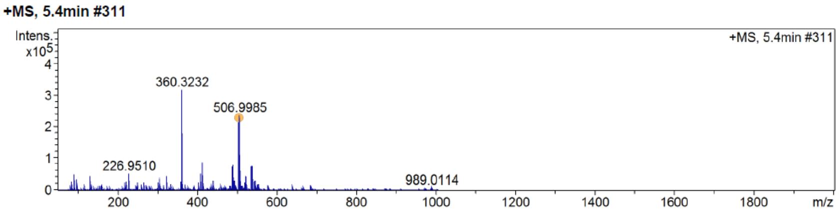

Meas. m/z	#	Ion Formula	m/z	err [ppm]	mSigma	# mSigma	Score	rdb	e⁻ Conf	N-Rule
505.0005	1	C23H19BrN2NaOS2	505.0014	1.9	11.4	1	100.00	14.5	even	ok

Figure S10. Mass spectra of **3S.**

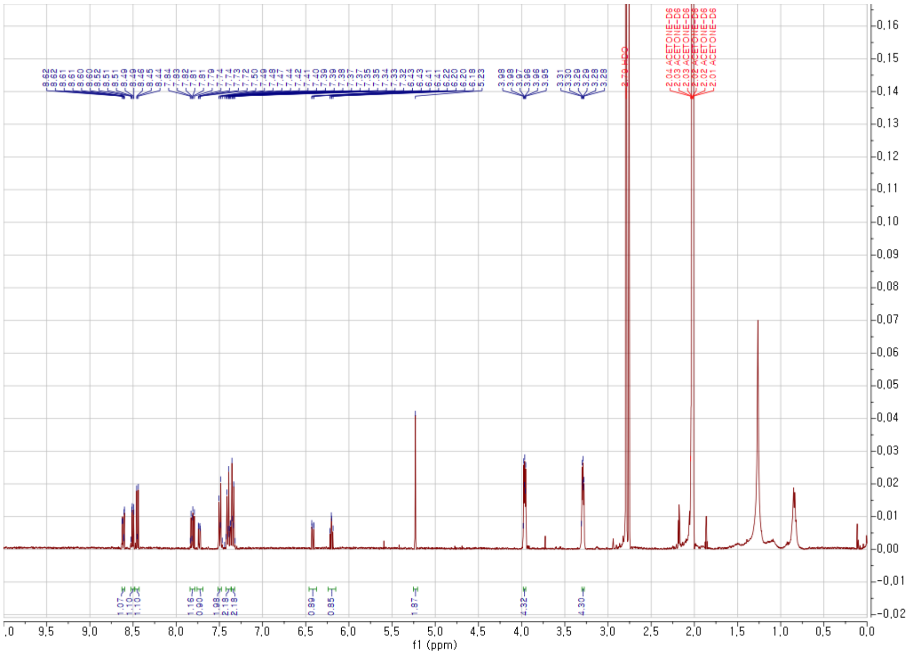

Figure S11. ^{1}H-NMR spectra of **4O** in Acetone-d_6.

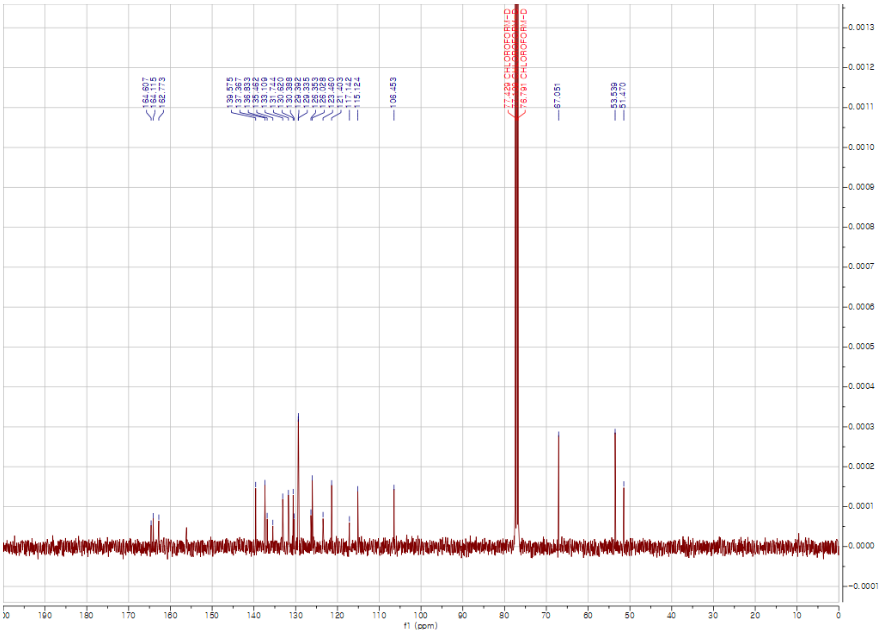

Figure S12. ^{13}C-NMR spectra of **4O** in $CDCl_3$.

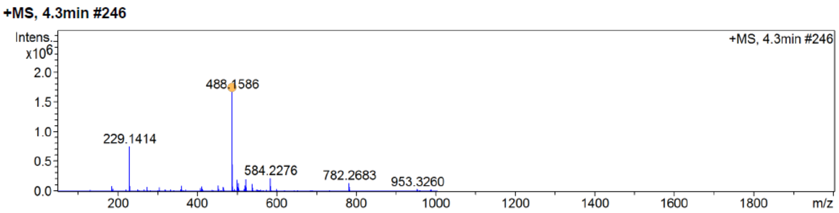

Meas. m/z	#	Ion Formula	m/z	err [ppm]	mSigma	# mSigma	Score	rdb	e⁻ Conf	N-Rule
488.1586	1	C28H23N3NaO4	488.1581	-1.0	23.1	1	100.00	18.5	even	ok
	2	C29H19N7Na	488.1594	1.7	39.8	2	41.89	23.5	even	ok

Figure S13. Mass spectra of **4O.**

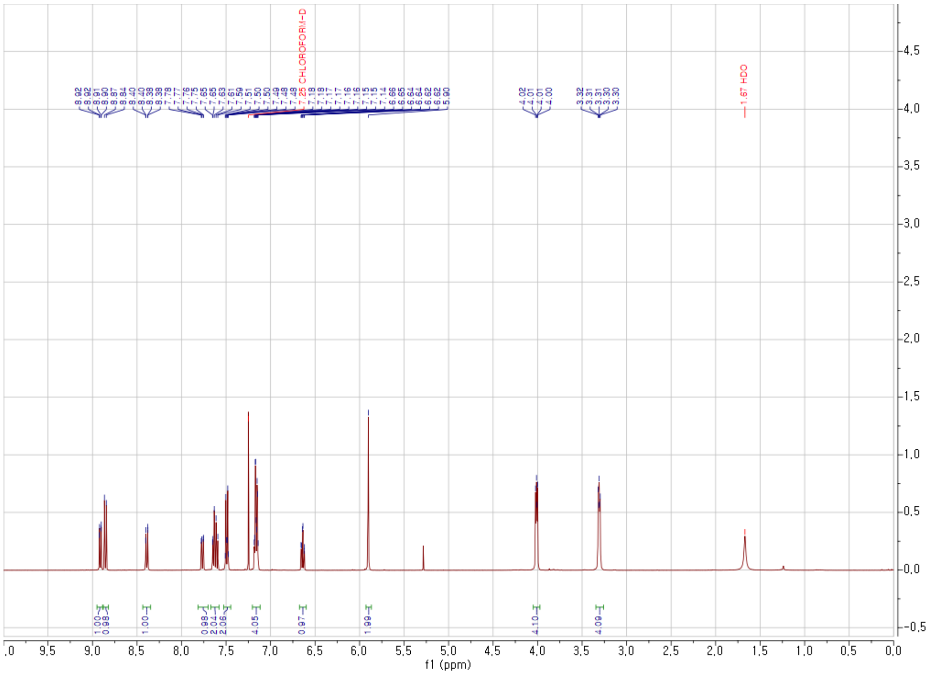

Figure S14. ^{1}H-NMR spectra of **4S** in $CDCl_3$.

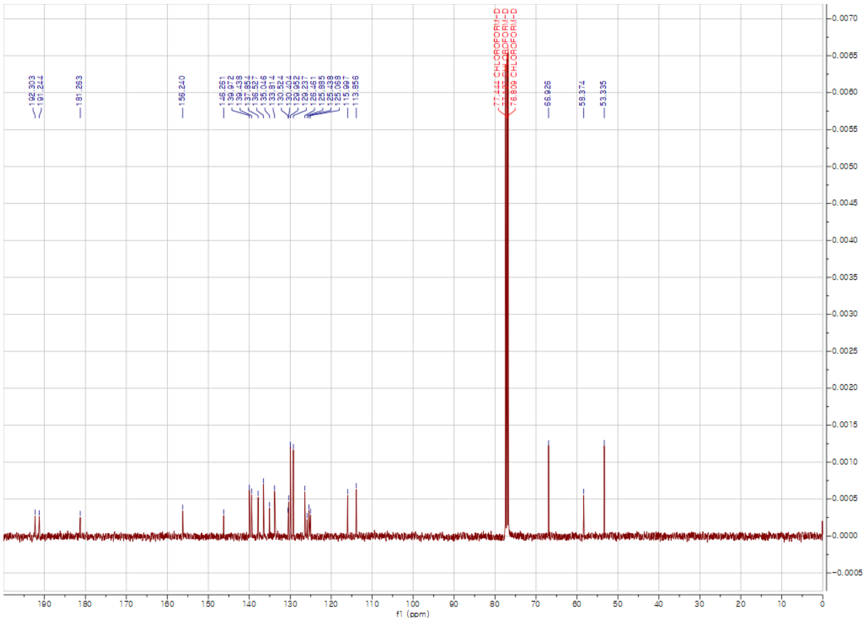

Figure S15. ^{13}C-NMR spectra of **4S** in $CDCl_3$.

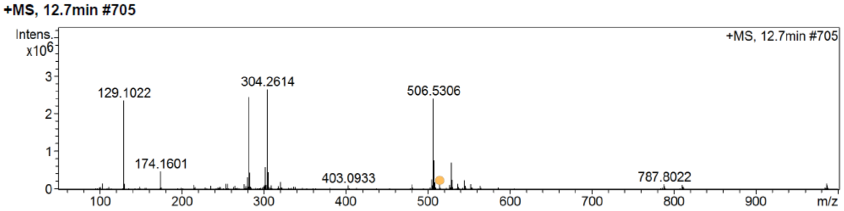

Meas. m/z	#	Ion Formula	m/z	err [ppm]	mSigma	# mSigma	Score	rdb	e⁻ Conf	N-Rule
514.1077	1	C28H24N3OS3	514.1076	-0.2	20.1	1	100.00	18.5	even	ok

Figure S16. Mass spectra of **4S.**

Table S1. Crystal data and structure refinement for **3O.**

	**3O**
Formula	$C_{23}H_{19}BrN_2O_3$
M_w	451.31
Cryst. sys.	Monoclinic
Space group	*Pn*
a (Å)	4.4070(9)
b (Å)	15.706(3)
c (Å)	27.517(6)
α (°)	90
β (°)	90.25(3)
γ (°)	90
V (Å^3)	1904.6(7)
Z	4
ρ (Mg/m^3)	1.574
μ (mm^{-1})	2.107
R_{int}	0.0180
GoF on F^2	1.026
R_1 [I>2$\sigma(I)$][a]	0.0355
wR_2 (all data)[b]	0.0984
CCDC deposition number	2168852

[a] $R_1 = \Sigma||F_o| - |F_c||/\Sigma|F_o|$, [b] $wR_2 = (\Sigma[w(F_o^2 - F_c^2)^2]/\Sigma[w(F_o^2)^2])^{1/2}$

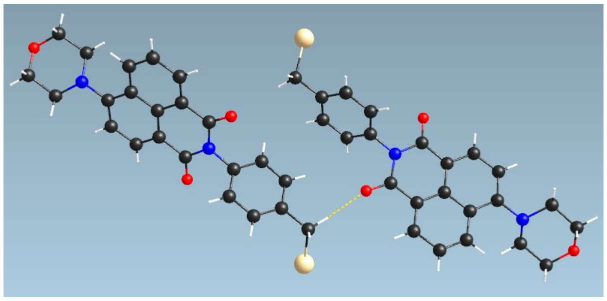

Figure S17. X-Ray crystal structures of **3O.**

3.2. Photophysical results

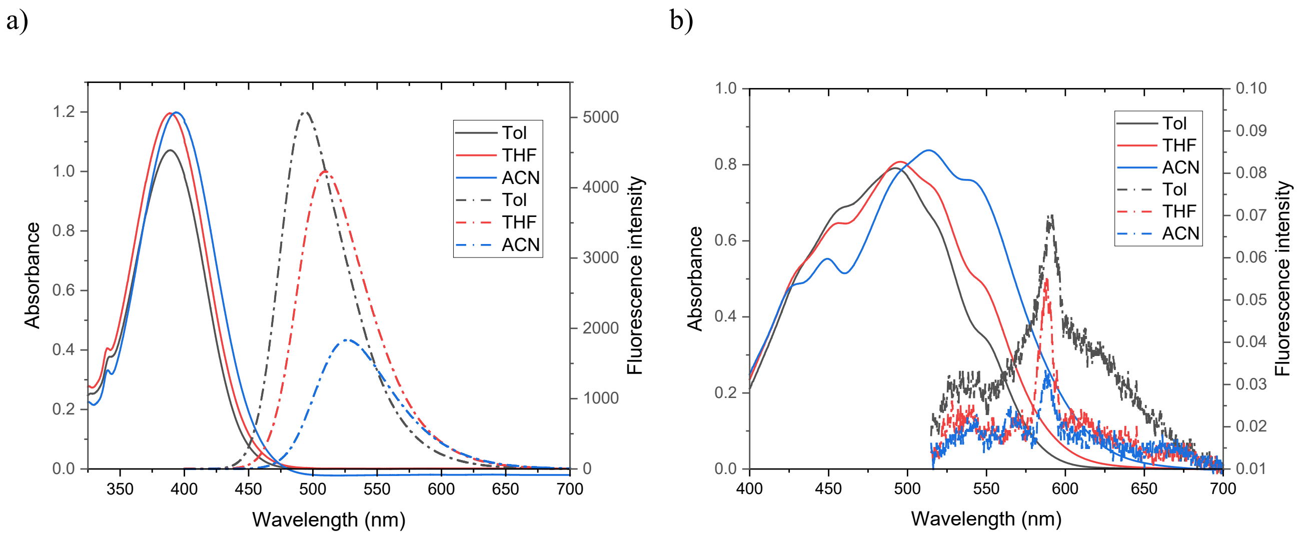

Figure S18. UV–Vis (solid line) and fluorescence emission (dash dot line) spectra of (a) **3O** (100 uM, λ_{ex} =390 nm, slit 5/5) and (b) **3S** (40 uM, λ_{ex} =500 nm, slit 5/5) in Tol, THF, and ACN.

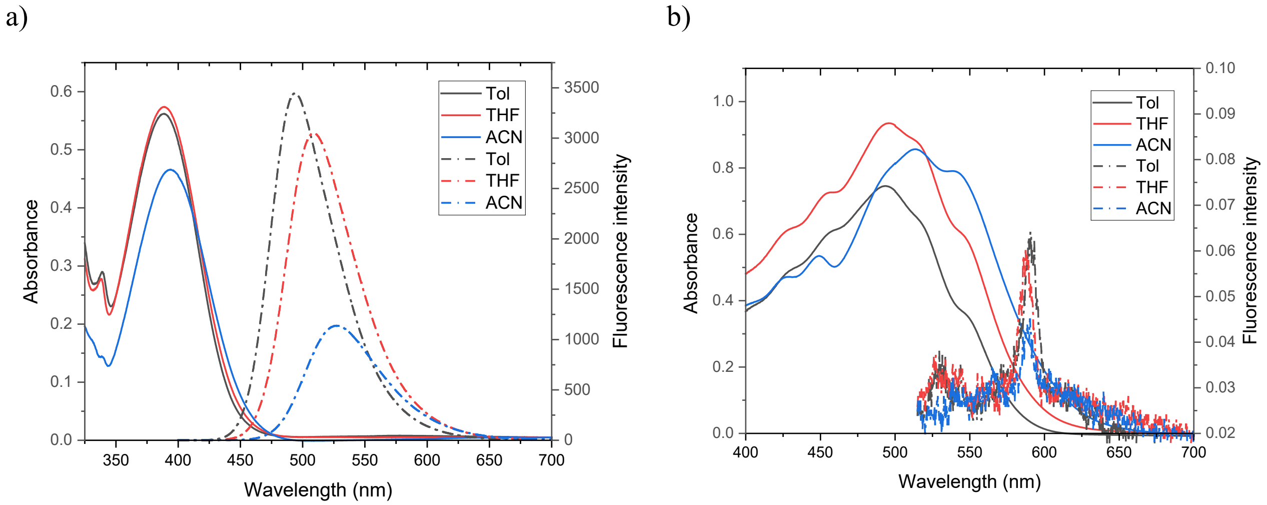

Figure S19. UV–Vis (solid line) and fluorescence emission (dash dot line) spectra of (a) **4O** (40 uM, λ_{ex} =390 nm, slit 5/5) and (b) **4S** (40 uM, λ_{ex} =500 nm, slit 5/5) in Tol, THF, and ACN.

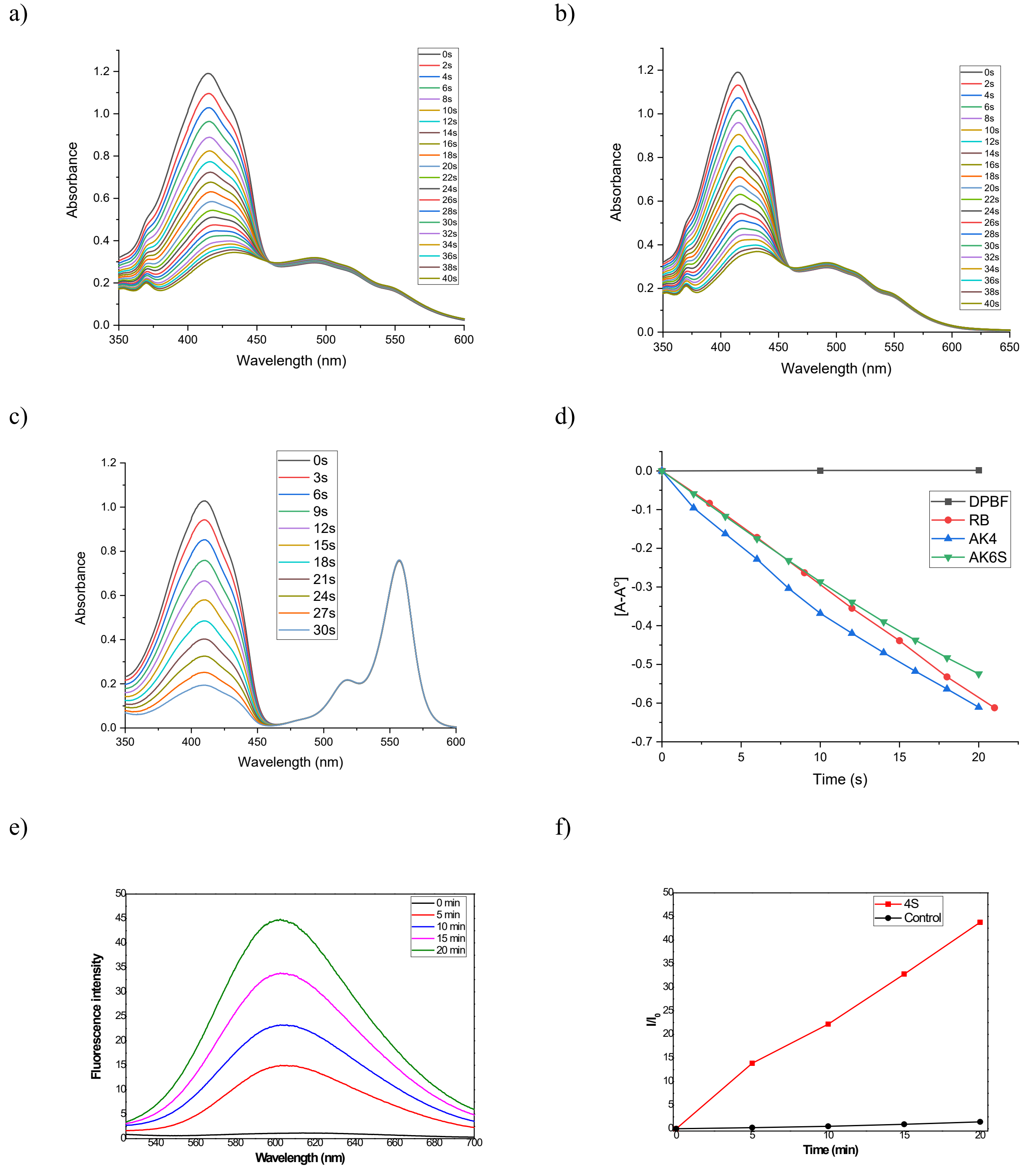

Figure S20. Time-dependent decrease in absorbance of DPBF (50 uM) in the presence of (a) **3S** (20 µM); (b) **4S** (20 µM); (c) **RB** (14 µM) (ref) in ACN during 0–40s (absorbance ~ 0.2); (d) comparison of decrease in absorbance of DPBF (50 uM). The samples were continuously irradiated by a green LED light source (530 nm, 5 mW cm^{-2}); (e) time-dependent increase of fluorescence emission of DHE (40 uM) (λ_{ex} = 510 nm) in the presence of **4S** (40 µM) in DW (10 %FBS) during 0–20 min; (f) comparison of increase in fluorescence emission of DHE (λ_{ems} = 600 nm). The samples were continuously irradiated by a green LED light source (530 nm, 5 mW cm^{-2}) and by a white light source (20 mW cm^{-2}).

a)

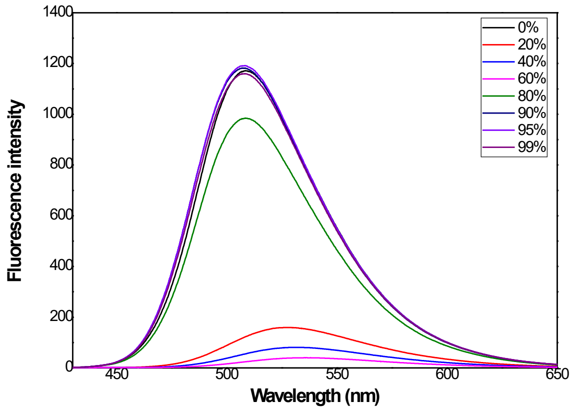

b)

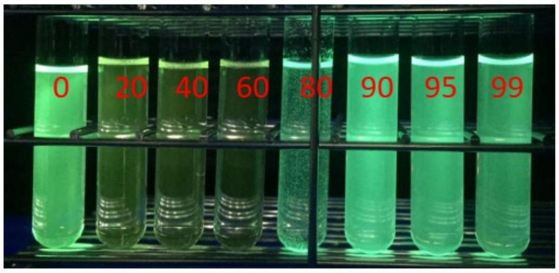

Figure S21. (a) Fluorescence emission spectra and (b) fluorescence image of **3O** in THF/DW (0-99%).

a)

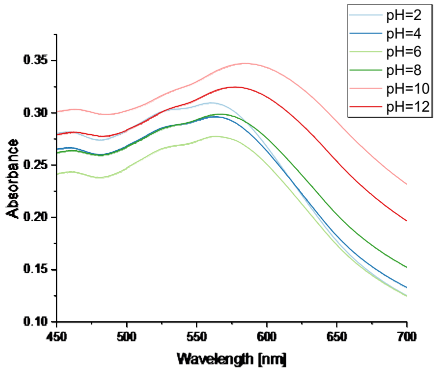

b)

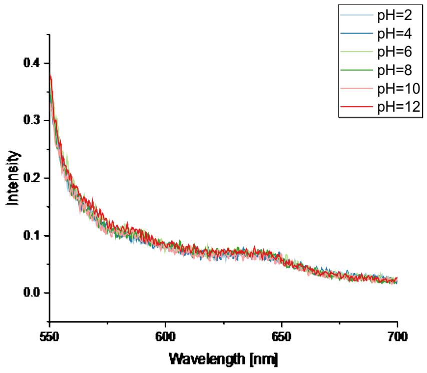

c)

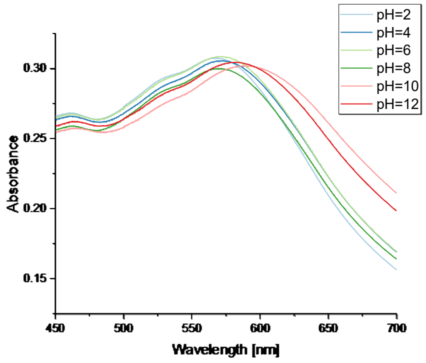

d)

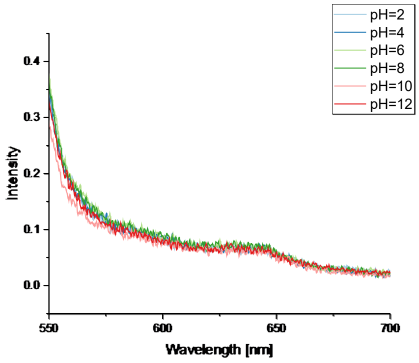

Figure S22. (a, c) UV–vis absorption and (b, d) FL spectra of **3S** and **4S** (20 mM), respectively, in THF/BR buffer (1/9) solution with various pH values (λ_{ex} = 530 nm, slit width 5/5).

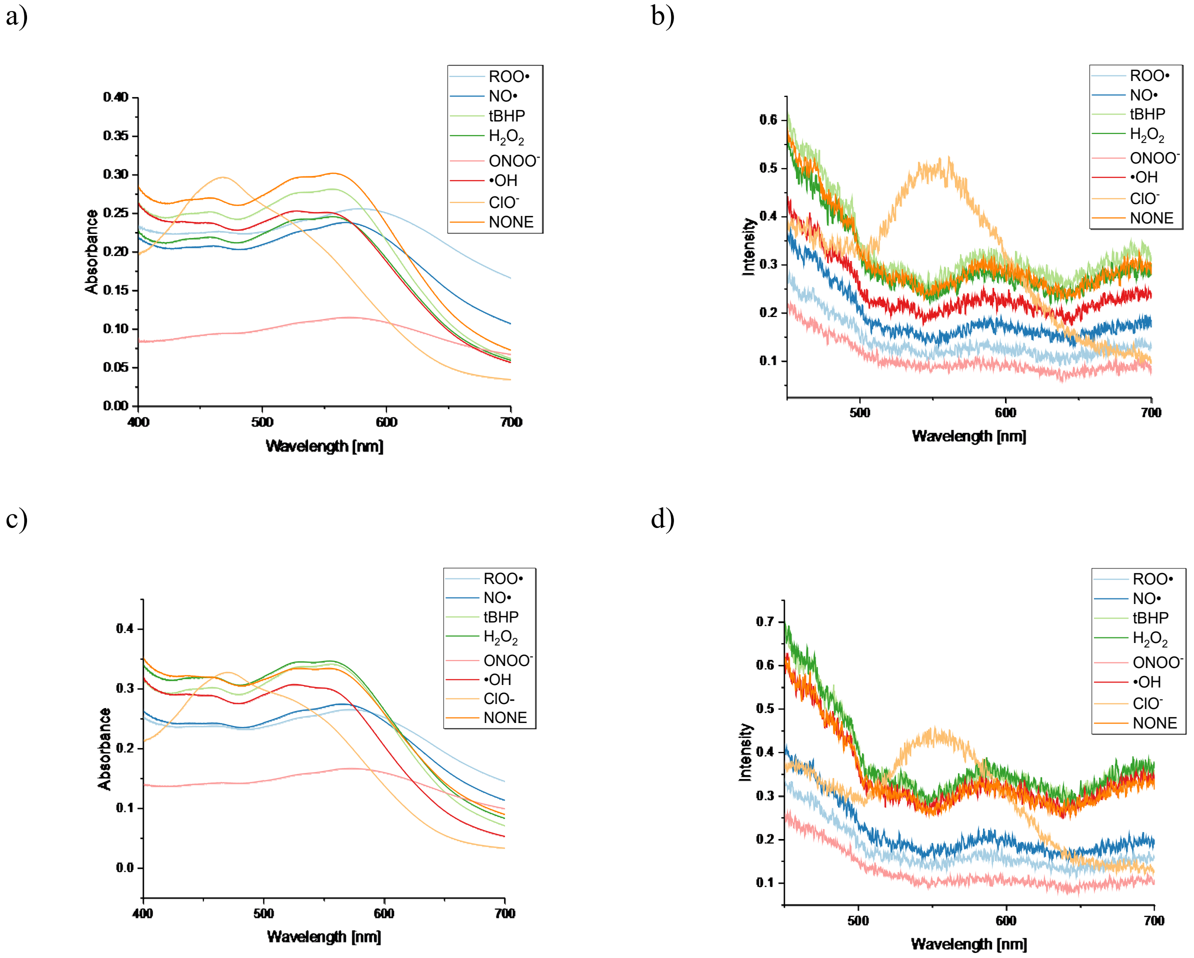

Figure S23. (a, c) UV–vis absorption and (b, d) FL spectra of **3S** and **4S** (20 mM), respectively, in THF/DW (1/9) solution with various ROS/RNS environments (λ_{ex} = 380 nm, slit width 5/5).

3.3. Computational calculation results

Table S2. Molecular orbitals and energies (eV) of **3O**, **3S**, **4O,** and **4S**.

	**3O**	**3S**	**4O**	**4S**
LUMO+2	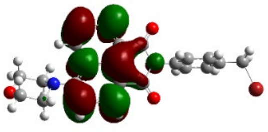 -1.18 eV	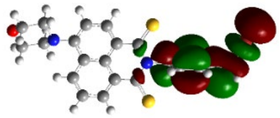 -1.31 eV	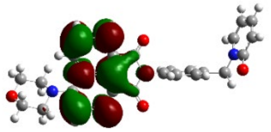 -1.14 eV	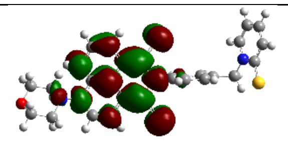 -1.65 eV
LUMO+1	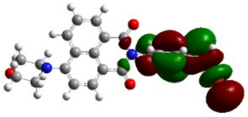 -1.30 eV	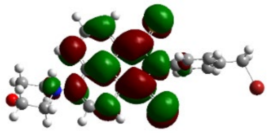 -1.66 eV	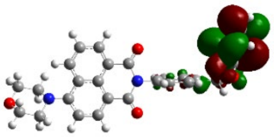 -1.37 eV	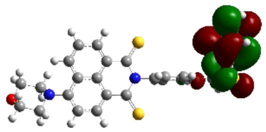 -1.71 eV
LUMO	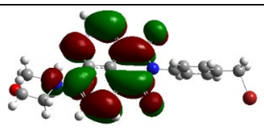 -2.68 eV	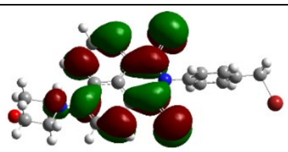 -3.23 eV	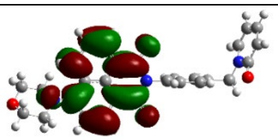 -2.63 eV	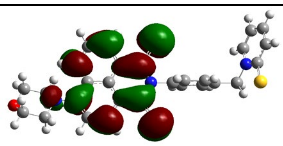 -3.22 eV
HOMO	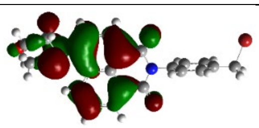 -6.26 eV	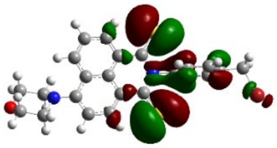 -5.70 eV	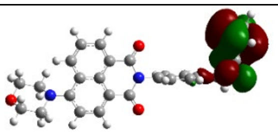 -6.03 eV	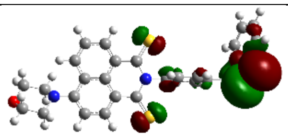 -5.51 eV
HOMO-1	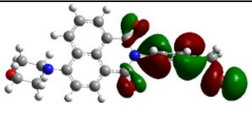 -6.92 eV	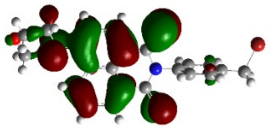 -6.05 eV	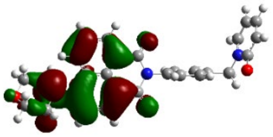 -6.22 eV	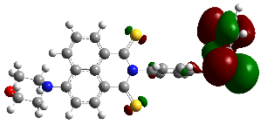 -5.53 eV
HOMO-2	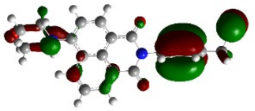 -7.17 eV	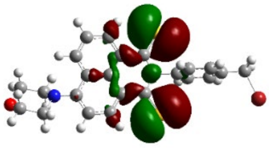 -6.34 eV	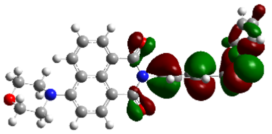 -6.83 eV	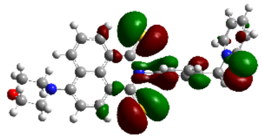 -5.73 eV
HOMO-3	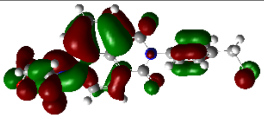 -7.27 eV	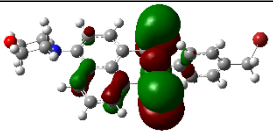 -6.56 eV	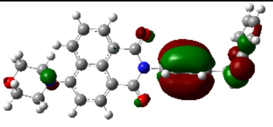 -7.08 eV	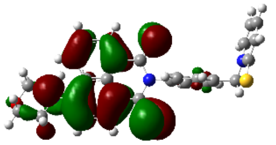 -6.04 eV

Table S3. Calculated electronic transition with significant oscillator strengths (*f*) of **3O**, **3S**, **4O,** and **4S.**

	**3O**	**3S**	**4O**	**4S**
**S_1**	**3.1749 eV**<br>f=0.3573<br>H → L 97.7%	1.8579 eV<br>f=0.0001	**3.1788 eV**<br>f=0.3612<br>H → L 97.7%	1.8614 eV<br>f=0.0001
**S_2**	4.0178 eV<br>f=0.0221	2.3889 eV<br>f=0.0479	3.6635 eV<br>f=0.0000	2.3859 eV<br>f=0.0681
**S_3**	4.0357 eV<br>f=0.0015	**2.5689 eV**<br>f=0.4369<br>[H – 2] → L 13.7%<br>[H - 1] → L 83.7 %	4.0160 eV<br>f=0.0200	**2.5656 eV**<br>f=0.4189<br>[H – 4] → L 19.1 %<br>[H - 3] → L 74.5 %
**S_4**	4.0783 eV<br>f=0.0526	3.3151 eV<br>f=0.0925	4.0364 eV<br>f=0.0009	2.5813 eV<br>f=0.0038
**T_1**	2.1629	1.6653	2.1629 eV	1.6644
**T_2**	3.2174	1.7340	2.8519 eV	1.7374
**T_3**	3.3435	2.2455	3.2223 eV	2.2484
**T_4**	3.5206	2.3368	3.4718 eV	2.3393
**T_5**	3.8417	2.8844	3.5247 eV	2.5733
**T_6**	3.8946	3.1553	3.6629 eV	2.5959

Table S4. Calculated ΔE_{ST}, spin–orbit coupling (SOC), and k_{ISC} of **3O** and **3S.**

	**3O**			**3S**		
	ΔE_{ST} (eV)	SOC (cm^{-1})	k_{ISC}	ΔE_{ST}	SOC	k_{ISC}
$S_1 \leftrightarrow T_1$	1.012	0.34967	7.6×10^{-7}	0.1926	68.39972	2.5×10^{12}
$S_1 \leftrightarrow T_2$	-	-	0	0.1239	6.36726	1.7×10^{10}
$S_1 \leftrightarrow T_3$	-	-	0	-	-	-
$S_2 \leftrightarrow T_1$	1.8549	11.77578	9.5×10^{-48}	0.7236	38.32926	1.2×10^{6}
$S_2 \leftrightarrow T_2$	0.8004	6.50042	547.6	0.6549	19.90450	9.0×10^{6}
$S_2 \leftrightarrow T_3$	0.6743	0.28873	791.5	0.1434	10.09644	4.7×10^{10}
$S_2 \leftrightarrow T_4$	0.4972	2.16556	3.4×10^{6}	0.1521	116.23555	6.5×10^{12}
$S_2 \leftrightarrow T_5$	0.2008	0.71062	2.7×10^{8}	-	-	-
$S_2 \leftrightarrow T_6$	0.1232	5.37187	1.1×10^{10}	-	-	-
$S_3 \leftrightarrow T_1$	1.8728	1.46648	8.1×10^{-51}	0.9036	9.97767	1.8
$S_3 \leftrightarrow T_2$	0.8183	0.99661	4.4	0.8349	59.39824	5.7×10^{3}
$S_3 \leftrightarrow T_3$	0.6922	0.04976	10.1	0.3234	32.15676	2.7×10^{11}
$S_3 \leftrightarrow T_4$	0.5151	0.39523	6.7×10^{6}	0.2321	26.12325	3.5×10^{11}
$S_3 \leftrightarrow T_5$	0.1940	3.14239	5.3×10^{9}	-	-	-
$S_3 \leftrightarrow T_6$	0.1411	0.86338	3.4×10^{8}	-	-	-
$S_4 \leftrightarrow T_1$	1.9446	1.12850	3.1×10^{-56}	1.6498	8.49496	1.4×10^{-34}
$S_4 \leftrightarrow T_2$	0.8901	1.77153	0.2	1.5811	69.22045	1.2×10^{-28}
$S_4 \leftrightarrow T_3$	0.7640	0.10718	1.2	1.0696	101.68783	5.8×10^{-4}
$S_4 \leftrightarrow T_4$	0.7569	0.28202	11.9	0.9783	0.96252	7.8×10^{-5}
$S_4 \leftrightarrow T_5$	0.2658	8.70790	3.3×10^{10}	0.4307	3.68749	5.5×10^{8}
$S_4 \leftrightarrow T_6$	0.2129	0.60513	1.9×10^{10}	0.1598	1.12297	6.3×10^{8}
$S_1 \leftrightarrow T_n$			7.6×10^{-7}			2.5×10^{12}
$S_2 \leftrightarrow T_n$			1.2×10^{10}			6.6×10^{12}
$S_3 \leftrightarrow T_n$			5.7×10^{9}			6.2×10^{11}
$S_4 \leftrightarrow T_n$			3.4×10^{10}			1.2×10^{9}
Sum			5.1×10^{10}			9.7×10^{12}

Table S5. Calculated ΔE_{ST}, spin–orbit coupling (SOC), and k_{ISC} of **4O** and **4S.**

	**4O**			**4S**		
	ΔE_{ST} (eV)	SOC (cm^{-1})	k_{ISC}	ΔE_{ST}	SOC (cm^{-1})	k_{ISC}
$S_1 \leftrightarrow T_1$	1.0159	0.34429	5.4×10^{-7}	0.1970	67.95918	2.5×10^{12}
$S_1 \leftrightarrow T_2$	0.3269	0.00446	4.9×10^{3}	0.1240	4.45451	8.1×10^{9}
$S_1 \leftrightarrow T_3$	-	-	-	-	-	-
$S_2 \leftrightarrow T_1$	1.5006	0.44292	1.8×10^{-28}	0.7215	37.34813	1.3×10^{6}
$S_2 \leftrightarrow T_2$	0.8116	0.02562	4.3×10^{-3}	0.6485	21.13613	1.4×10^{7}
$S_2 \leftrightarrow T_3$	0.4412	0.30343	2.9×10^{6}	0.1375	13.65121	8.3×10^{10}
$S_2 \leftrightarrow T_4$	0.1917	0.07513	3.0×10^{6}	0.0466	115.35787	2.3×10^{12}
$S_2 \leftrightarrow T_5$	0.1388	0.09129	3.7×10^{6}	-	-	-
$S_2 \leftrightarrow T_6$	0.0006	0.01719	2.3×10^{4}	-	-	-
$S_3 \leftrightarrow T_1$	1.8531	11.72117	1.3×10^{-47}	0.9012	7.50242	1.2
$S_3 \leftrightarrow T_2$	1.1641	0.00675	5.5×10^{-16}	0.8282	35.81236	3.1×10^{3}
$S_3 \leftrightarrow T_3$	0.7937	6.49615	8.1×10^{2}	0.3172	20.87235	1.2×10^{11}
$S_3 \leftrightarrow T_4$	0.5442	0.31135	1.6×10^{5}	0.2263	18.24240	1.7×10^{11}
$S_3 \leftrightarrow T_5$	0.4913	2.17775	4.1×10^{7}	-	-	-
$S_3 \leftrightarrow T_6$	0.3531	1.38326	3.3×10^{8}	-	-	-
$S_4 \leftrightarrow T_1$	1.8735	1.07637	3.9×10^{-51}	0.9169	9.09848	0.6
$S_4 \leftrightarrow T_2$	1.1845	0.00427	3.2×10^{-17}	0.8439	45.37659	1.9×10^{3}
$S_4 \leftrightarrow T_3$	0.8141	0.75848	3.3	0.3329	21.83444	1.1×10^{11}
$S_4 \leftrightarrow T_4$	0.5646	0.02927	7.2×10^{2}	0.2420	24.31005	2.9×10^{11}
$S_4 \leftrightarrow T_5$	0.5117	0.29687	4.2×10^{5}	0.0008	4.97140	1.9×10^{9}
$S_4 \leftrightarrow T_6$	0.3735	0.20130	5.0×10^{6}	-	-	
$S_1 \leftrightarrow T_n$			4.9×10^{3}			2.5×10^{12}
$S_2 \leftrightarrow T_n$			9.7×10^{6}			2.4×10^{12}
$S_3 \leftrightarrow T_n$			3.7×10^{8}			2.9×10^{11}
$S_4 \leftrightarrow T_n$			5.5×10^{6}			4.1×10^{11}
Sum			3.9×10^{8}			5.6×10^{12}

3.4. Cell experiment results

a)

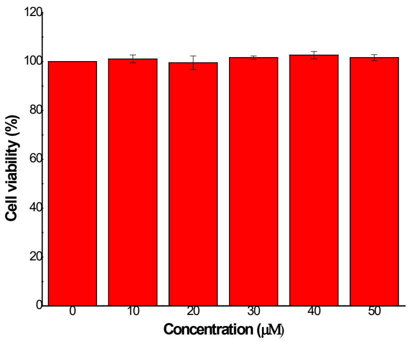

c)

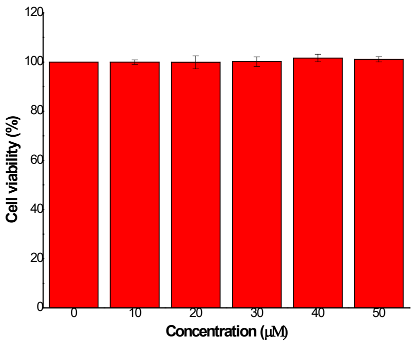

b)

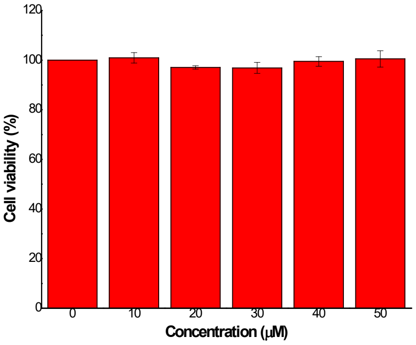

d)

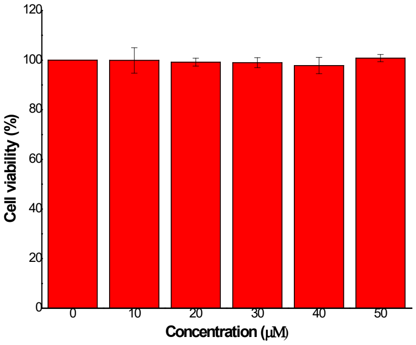

Figure S24. Cell viability of Hela Cells in the presence of a) **3O**; b) **3S**; c) **4O**; and d) **4S** (0 -50 μM).

a) b) c) d)

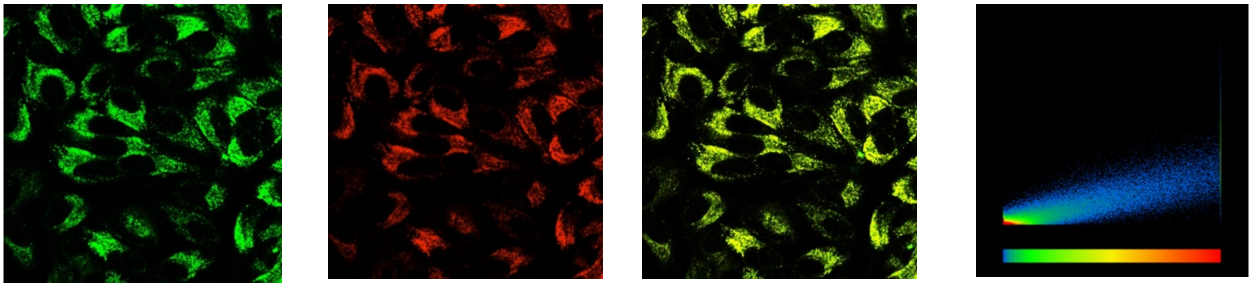

Figure S25. Hela cells costained with (a) 10 μM **3O** (λ_{ex} = 405 nm, λ_{em} = 600 nm) for 30 min and (b) 50 nM LysoTracker deep red for 1 h (λ_{ex}= 647 nm, λ_{em} = 655 nm) in PBS and fluorescence images acquired by confocal microscopy. (c) An overlay. (d) Colocalization analysis plot of the LysoTracker and **3O**. Pearson's overlap coefficient, R_P = = 0.85.

a) b) c) d)

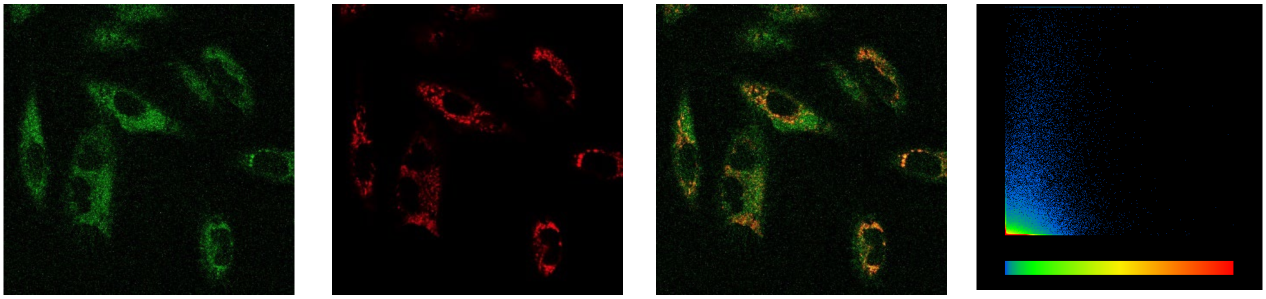

Figure S26. Hela cells costained with (a) 10 μM **3O** (λ_{ex} = 405 nm, λ_{em} = 600 nm) for 30 min and (b) 100 nM MitoTracker deep red for 45 min (λ_{ex} = 644 nm, λ_{em} = 665 nm) in PBS and fluorescence images acquired by confocal microscopy. (c) An overlay. (d) Colocalization analysis plot of the MitoTracker and **3O**. Pearson's overlap coefficient, R_P = 0.29.

a) b) c) d)

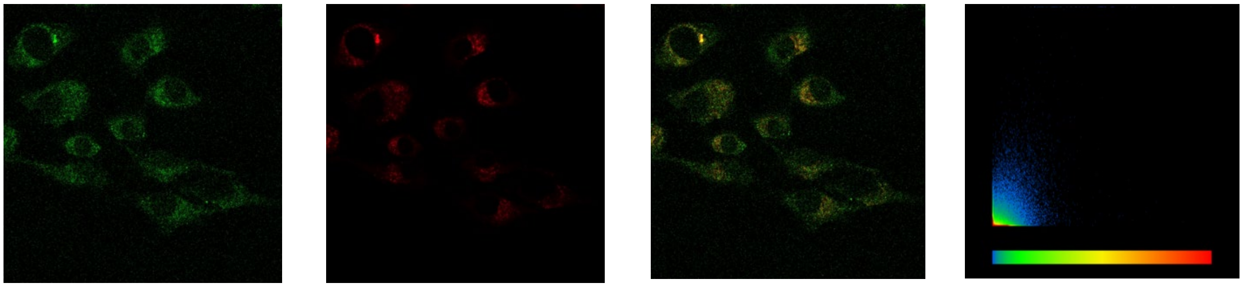

Figure S27. Hela cells costained with (a) 10 μM **4O** (λ_{ex} = 405 nm, λ_{em} = 600 nm) for 30 min and (b) 100 nM MitoTracker deep red for 45 min (λ_{ex} = 644 nm, λ_{em} = 665 nm) in PBS and fluorescence images acquired by confocal microscopy. (c) An overlay. (d) Colocalization analysis plot of the MitoTracker and **4O**. Pearson's overlap coefficient, R_P = 0.37.

4. Reference

1. Dai, H., *et al.* Selective and Sensitive Fluorescent Chemosensors for Cu2+ Ion Based upon Bis-1,8-naphthalimide Dyads. *Chinese Journal of Chemistry* **2012,** *30*, 267.
2. Brouwer, A. M. Standards for photoluminescence quantum yield measurements in solution (IUPAC Technical Report). *Pure and Applied Chemistry* **2011,** *83*, 2213.
3. Üçüncü, M., *et al.* BODIPY–Au(I): A Photosensitizer for Singlet Oxygen Generation and Photodynamic Therapy. *Organic Letters* **2017,** *19*, 2522.
4. Durantini, A. M., *et al.* Reactive Oxygen Species Mediated Activation of a Dormant Singlet Oxygen Photosensitizer: From Autocatalytic Singlet Oxygen Amplification to Chemicontrolled Photodynamic Therapy. *Journal of the American Chemical Society* **2016,** *138*, 1215.
5. Gao, X., *et al.* Evaluation of Spin-Orbit Couplings with Linear-Response Time-Dependent Density Functional Methods. *Journal of Chemical Theory and Computation* **2017,** *13*, 515.
